# An ethnopharmacological review on the therapeutical properties of flavonoids and their mechanisms of actions: A comprehensive review based on up to date knowledge

**DOI:** 10.1016/j.toxrep.2022.03.011

**Published:** 2022-03-14

**Authors:** Doha H. Abou Baker

**Affiliations:** Medicinal and Aromatic Plants Dept., Pharmaceutical and Drug Industries institute, National Research Centre, Cairo, Egypt

**Keywords:** Flavonoids, Structure, Therapeutical activities, Mechanism of action, Structure activity relationship

## Abstract

Flavonoids -a class of low molecular weight secondary metabolites- are ubiquitous and cornucopia throughout the plant kingdom. Structurally, the main structure consists of C6-C3-C6 rings with different substitution patterns so that many sub-classes are obtained, for example: flavonols, flavonolignans, flavonoid glycosides, flavans, anthocyanidins, aurones, anthocyanidins, flavones, neoflavonoids, chalcones, isoflavones, flavones and flavanones. Flavonoids are evaluated to have drug like nature since they possess different therapeutic activities, and can act as cardioprotective, antiviral, antidiabetic, anti-inflammatory, antibacterial, anticancer, and also work against Alzheimer's disease and others. However, information on the relationship between their structure and biological activity is scarce. Therefore, the present review tries to summarize all the therapeutic activities of flavonoids, their mechanisms of action and the structure activity relationship.

## Highlights

• Latest updated ethnopharmacological review of the therapeutic effects of flavonoids.
• Flavonoids are attracting attention because of their therapeutic properties.
• Flavonoids are valuable candidates for drug development against many dangerous diseases.
• This overview summarizes the most important therapeutic effect and mechanism of action of flavonoids.
• General knowledge about the structure activity relationship of flavonoids is summarized.
• Substitution of chemical groups in the structure of flavonoids can significantly change their biological and chemical properties.
• The chemical properties of the basic flavonoid structure should be considered in a drug-based structural program.

## Graphical Abstract

**Figure:**
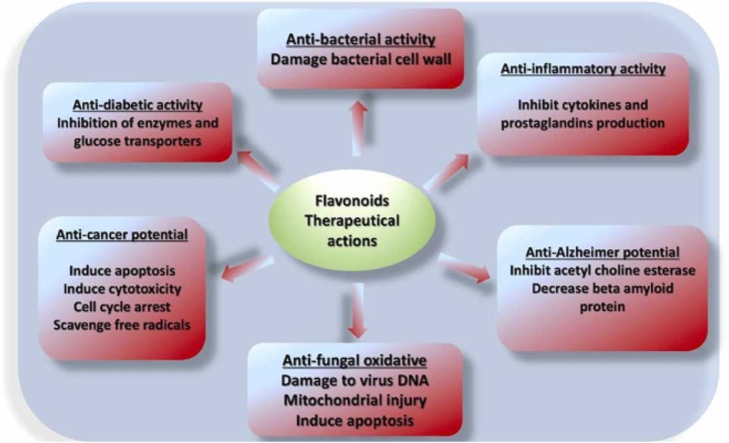

## 1 Introduction

Recent studies suggest the rational development of more potent, less toxic compounds that can be used clinically to treat of patients suffering from chronic diseases that cause oxidative stress.

Phytochemicals are plant-based molecules that protect people from many chronic diseases. Flavonoids are one of the most exciting types of phenolic compounds. They are found in a wide variety of plants. Studies in the chemistry of natural products are very common in leaves, flower tissues, pollen and fruits. This phytocompound is also abundant in stem and bark, and represents an integral part of human healthy life style. Flavonoids are existed broadly in nature. Concerns about their extensive profitable bioactive benefits, including anti-inflammatory, antioxidant, anti-viral, antifungal, antibacterial, antihypertensive, cardioprotective, anti-ulcer, anti-diabetic, anti-Alzheimer, anti-depression, and anti-cancer effects have been receiving great attention and support by numerous studies. Till now, more than 9000 flavonoids have been reported, and their daily intake varies between 20 mg and 500 mg, mainly from dietary supplements including apples, grapes, berries, tea, tomatoes and onions.

Notably, despite their broad benefits and wide distribution, flavonoids have poor bioavailability, which can significantly influence their nutritional value. Besides, information on their pharmacokinetics is limited. How the problem can be fixed is far from being resolved. This review attempts to summarize all the data about structure and activity of flavonoids, with particular emphasis on their mechanism of action.

## 2 Structure of flavonoids

**Fig. 1:**
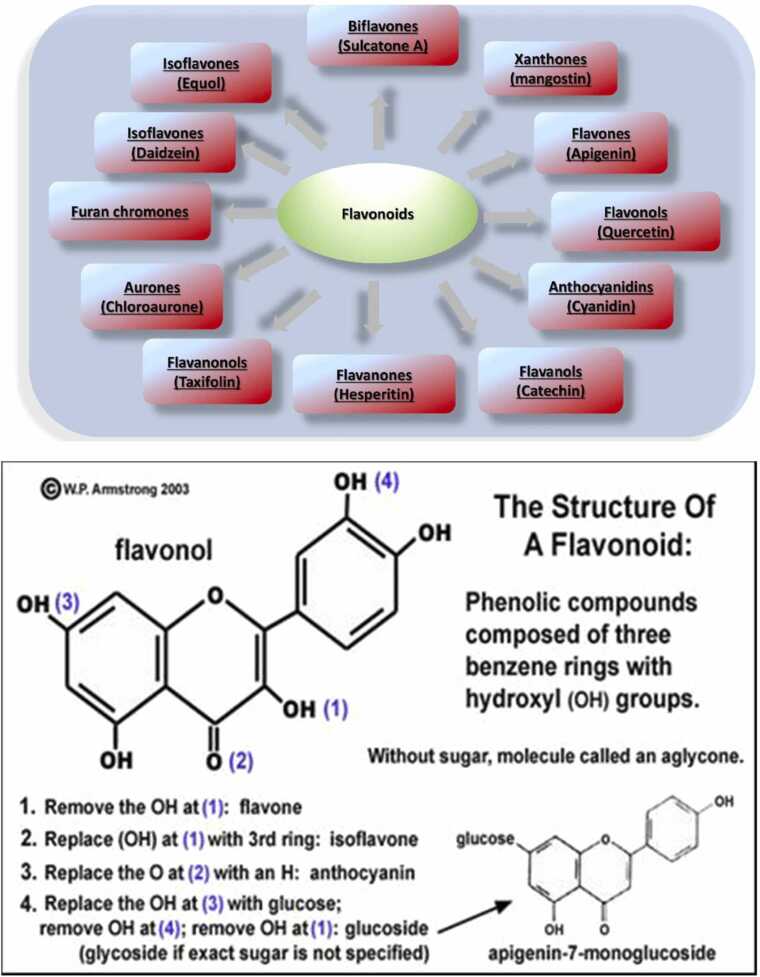
Flavonoids subclasses and their representative flavonoids.

Flavonoids are divided into several classes. They have a C_6_C_3_C_6_ structure consisting of two aromatic rings together with a heterocyclic oxygenated benzopyran ring (Fig. 1).

## 3 Therapeutical potential of flavonoids

**Fig. 2:**
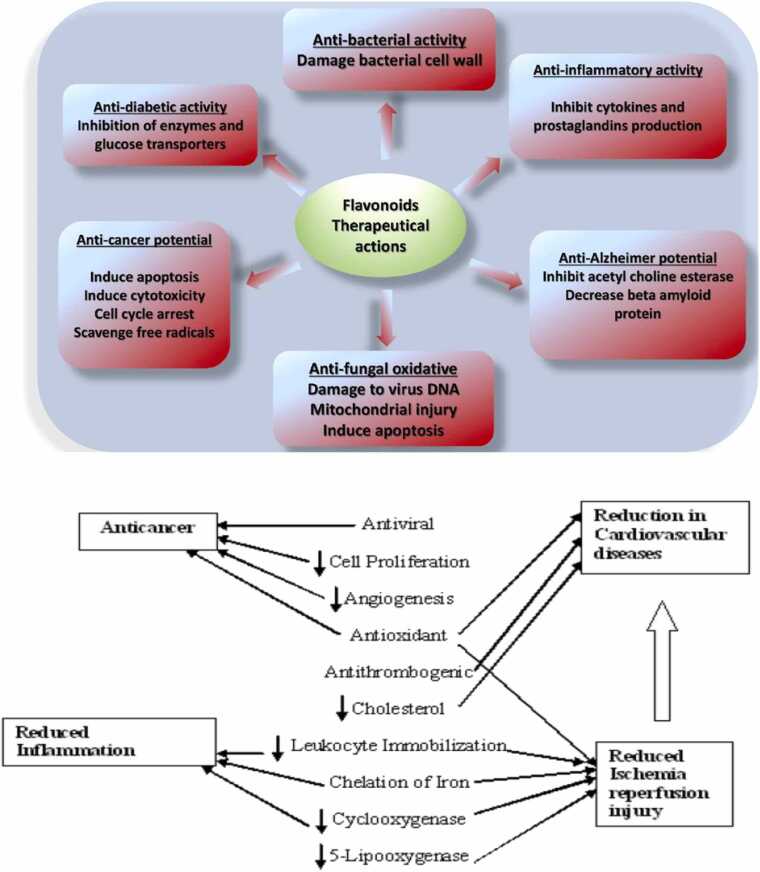
A) Flavonoid therapeutical actions. B) Effects of flavonoids on many diseases.

Flavonoids (phenolic compounds) are of the prevalent secondary metabolites in plants with about 9000 different compounds [280] being biologically active (Fig. 2). Due to differences in the structure, distribution, metabolism and bioavailability of flavonoids, different flavonoids can have different effects on human health [10], [101], [102], [184], [230], [3], [4], [5], [6], [66], [67], [68], [7]. In order to delineate the therapeutic activities of flavonoids more in depth, mode of flavonoids action and structure activity relationship were comprehensively reviewed.

### 3.1 Potential against Alzheimer's disease

Flavonoids are reported to have strong therapeutic activity in the treatment of Alzheimer's disease and are considered future drug candidates. The report included in this comprehensive review suggests that the main mechanism of action in the treatment of Alzheimer's disease is decreased due to the production of Reactive Oxygen Species (ROS) and beta amyloid protein. About 127 flavonoids were tested for anti-Alzheimer's activity and showed acetyl and butylcholinesterase inhibitors were responsible for their activity.

#### 3.1.1 Anti-Alzheimer mechanism of action

**Fig. 3:**
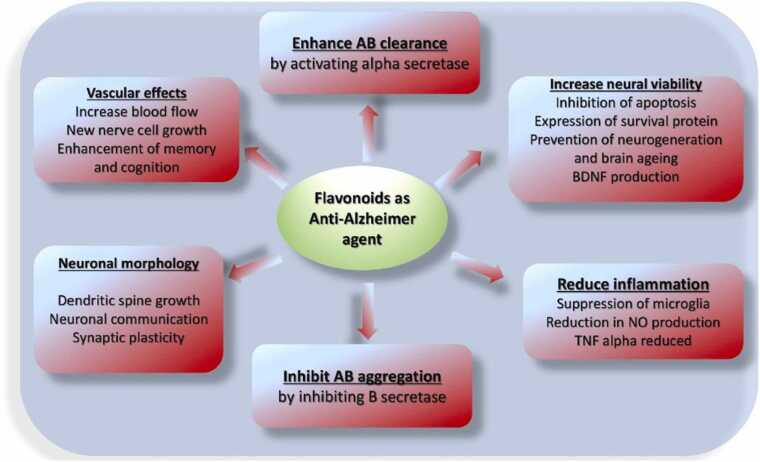
Flavonoid mechanism of anti-Alzheimer activity.

Flavonoids can reduce Aβ plaque either by increasing the activity of α-secretase or by inhibiting β-secretase activity. They can interfere with fibrillation, inhibit beta amyloid protein aggregation through metal chelating activity, increase cerebral vascular blood flow, decrease beta amyloid protein levels, or inhibit the factors involved in nerve damage, for example: ROS, Nitric Oxide (NO), beta amyloid protein, phosphorylation of tau and Acetyl Choline Esterase (AChE) as summarized in Fig. 3 and Table 1.

**Table 1:** List of flavonoids with anti-Alzheimer effect and their mechanism of action.

Flavonoids	Mechanism of action	References
Hesperidin	Promotes neural differentiation	[7]
	Decrease β amyloid plaques	
	Inhibit AChE	
Anthocyanin	Decrease β amyloid protein	Vepsalane et al., 2013
Nariginin	Suppress neuronal death	Hernandez-Mantes et al., 2006
Silibinin	Suppress inflammatory response	[246]
	Decrease in ROS production	
Quercetin	Suppress apoptosis	Lee et al., 2003
	Increase AMPK activity	
	Down regulation of tau phosphorylation	
Baicalein	Increase dopaminergic level	[105]
		[281]
Resveratrol	Increase BDNF production	[298]
	Inhibit AChE	
Luteolin	Decrease Aβ plaque formation	Rezai-zadeh et al., 2009
Genistein	Increase neural survival	Weinreb et al., 2009
	Decrease apoptosis	
	Decrease Aβ plaque formation	
Myrecetin	Inhibit butylcholinesterase activity	Leclerc et al., 2001

#### 3.1.2 Structure activity relationship for anti-Alzheimer activity

Central Nervous System drugs require greater liposolubility that can be enhanced by non-polar fragments (ex: aliphatic rings, alkyls and halogen atoms) in the molecules. At the same time, topological polarity surface area can affect the cellular drug molecules penetration. Previous studies have shown that flavonoids contain lower topological polarity surface area and higher water-lipid partition coefficient that can bypass blood brain barrier with potential activity.

**Fig. 4:**
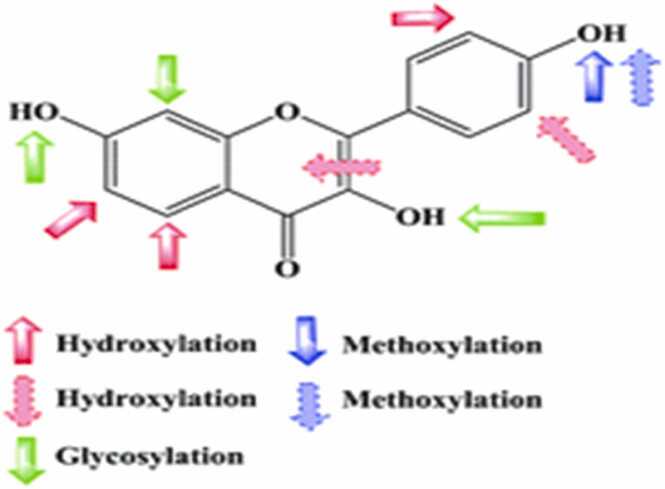
Summary of anti-Alzheimer structure activity relationships of flavonoids.

Xie et al. [284] examined the structural aspects of the AChE inhibitory potential of flavonoids and found that the OH group in the A ring [122] (Fale et al., 2012) and hydrogen bonding play a role in increasing affinity for AChE. AChE inhibition generally increases by flavones and flavonols. Whereas methoxylation, glycosylation and hydrogenation of the C_2_-C_3_ double bond decrease (Fig. 4). AChE inhibition depends on conjunction site, flavonoid class and sugar moiety.

### 3.2 Potential against depression

**Fig. 5:**
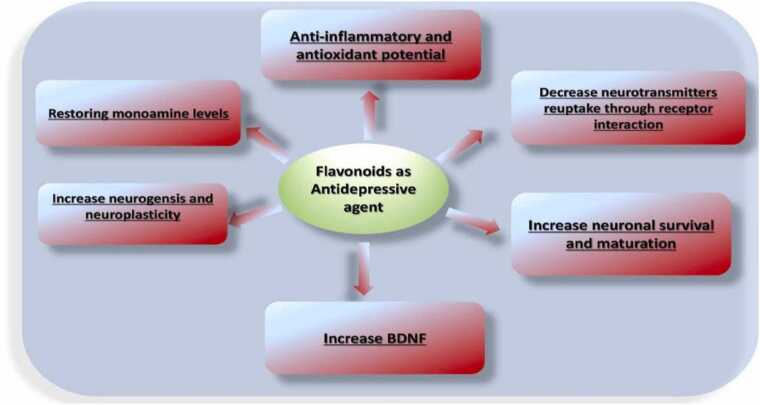
Flavonoid mechanism of antidepressant activity.

Flavonoids have been reported to have antidepressant activity [124], [25]. Updated reports suggest that apigenin exhibits antidepressant activity via dopaminergic mechanism [292], whilst luteolin reduces stress on endoplasmic reticulum [107]. Other studies indicate that icarin inhibits the NF-κB receptor and activation of the 3-inflammatory / caspase-1 / IL-1β axis in the hippocampus [153], whereas antidepressant activity of rutin is displayed by increasing monoamines in synaptic clefts (Nöldner and, 2002) (Fig. 5, Table 2).

**Table 2:** List of flavonoids with antidepressant effect and their mechanism of action.

Flavonoids	Mechanism of action	References
Kaempferol	Inhibit monoamine oxidase	[245]
Chrysin		[144]
Quercetin, quercetrin		[93]
Catechin and epicatechin		[99]
Isoflavone formononetin		Zhu et al., 2008
Baicalin		
Quercetin-3-O-apiosy1(1→2)-rhamnosy1(1→6) glucoside	Protect nerve cells	[147]
Rutin	Increase synthesis of noradrenaline or serotonin	Nöldner M, 2002
Apigenin	Inhibit monoamines	Nakazawaet al., 2003
Kaempferol	Decrease dopamine, serotonin, and norepinephrine	[146]
Isorhamnetin		[203]
Icariin	Improve abnormalities	[198]
Naringenin	Increase NA, GR and 5-HT levels in hippocampus	[290]
	Reduce serum corticosterone	
Astilbin	ActivateBDNF signaling pathway	[161]
	Up-regulate monoaminergic neurotransmitters	
Amentoflavone	Interact with 5-HT2 receptor and adrenoceptors	Ishola et al., 2012
	Ionotropic GABA receptor.	
Hyperoside A	Increase expression of BDNF	[302]
Hesperidin	Interact with 5-HT receptor.	[247]
Luteolin	Increase potency of GABAA receptor ion channel complex	[60]
Nobiletin	Interact with the noradrenergic, serotonergic, and dopaminergic systems.	[291]

#### 3.2.1 Structure-activity relationship

In flavonoids, the position of the OH group on ring A affects the antidepressant activity where compounds with the OH group at the 2,4 positions show high activity well as the C-glucoside flavones [77]. It has been reported that the sequence of antidepressant activity of flavonoids as follow: flavones >flavonols >flavonoids glycosides >flavanonols [85].

#### 3.2.2 Anti-depressant mechanism of action

The antidepression mechanism of flavonoids include a) restoring monoamine levels, b) increasing neural survival and maturation, c) increasing neurogensis and neuroplasticity, d) increasing BDNF, e) decreasing neurotransmitters reuptake through receptor interaction.

1. Flavonoids increase biogenic amines
  Flavonoids can increase levels of the monoamine neurotransmitter in neuronal synaptosomes, which leads to a reduction in clinical symptoms of depression [303], [304].
2. Inhibition of bioamine reuptake
  Flavonoids can re-absorbe 5-HT prevention by decreasing the number of 5-HT receptors and by inhibiting catechic acid transmethylase activity using synaptosomes [299]. This effect in turn induces the expression of neuroamine transmission in the brain [275].
3. Effects of flavonoids on the neuroendocrine system

Flavonoids can enhance 5-HT neurological function and the action of adenylate cyclase and neurotrophic factor 5-HT receptor mediated (Butterweck et al., 2000). The increase in phosphorylated BDNF and cAMP- response element binding protein (CREB) was caused by hippocampal nerve synthesis (Knorr et al., 2017). In addition, increase the hippocampal nerve synthesis and BDNF expression (An et al., 2011). Flavonoids also inhibit stress hormone levels and increase the expression of glucocorticoid receptors in the hippocampus and prevent PC12 nerve cell damage (Patil et al., 2014) as well as its ability for restoration of IL-6 and TNF-α in serum (Pan 2006).

Flavonoids can inhibit ACh and triphosadenine,and limit ATP and α-amino-3-OH-5-methanoic acid [36]. One possible associated mechanism includes restoration of the activity of COX-2 (Li et al., 2013a, 2013b). Additionally, flavonoids can decrease levels of corticosterone and adrenocorticotropic hormones and can regulate corticotropin-releasing factor mRNA expression because they can modulate the DNA binding activity of glucocorticoid and cAMP receptors as well as the phosphorylation of extracellular kinase signal in the hypothalamus region.

### 3.3 Antioxidant potential

Oxidative stress refers to the excessive production of free radicals and other highly active enzymes causing imbalance of intracellular antioxidant capacity, which lead to lipid peroxidation, protein denaturation, and DNA damage. Oxidative stress is one of the main signs of inflammation. However, prolonged oxidative stress can damage the surrounding molecules. Recent clinical studies have shown that oxidative stress plays a crucial role in the development of many dangerous diseases such as cardiovascular disease [218], [282], Alzheimer [234], [301], [41], cancer [189], [87], [9], diabetes [18]. The antioxidant potential of flavonoids has been well described in many studies (Havsteen 2002) [210].

#### 3.3.1 Mechanism of antioxidant action

The antioxidant capacities of flavonoids are much powerful than those of VitC and VitE [209] by the following mechanisms:(a) Mitigateoxidation caused by NO [262]. b) Metal chelating activity [70]. c) Inhibit oxidases [52]. d) Activate antioxidant enzymes [187]. e) Reduce α-tocopheryl radicals [89], [92]. f) Scavenge of ROS [187]. g) Increase in antioxidant properties of low molecularantioxidants [288]. h) Increase in uric acid levels [157].

The antioxidant effects of flavonoids also include a) inhibiting ROS production, either by chelating the trace elements or by inhibiting enzymes involved in ROS production; b) and improving regulation and protection of antioxidants. Flavonoids also inhibit ROS production enzymes, including monooxygenase, mitochondrial succinic oxidase, glutathione S-transferase, and NADH oxidase. The antioxidant mechanisms of flavonoids are listed in Table 3.

**Table 3:** Mechanisms of antioxidant activity of flavonoids.

Responsible structural elements	Mechanisms of antioxidant activity	References
4-(-C = O) group in conjugation with 3-OH group	Metal chelating activity	[207]
4-(-C = O) group with 5-OH group		
3′,4′-OH groups		
3′,4′-OH groups	Scavenge Peroxynitrite	[210]
3-OH group		
Flavones structure	Inhibit protein kinase C	[210]
7-OH group		
3′,4′-OH groups		
3′,4′-OH groups	Scavenge ROS	[207]
3,5,7-OH groups		
4-(-C = O) group in conjugation with 2,3-double bond		

#### 3.3.2 Structure activity relationship for antioxidant activity

**Fig. 6:**
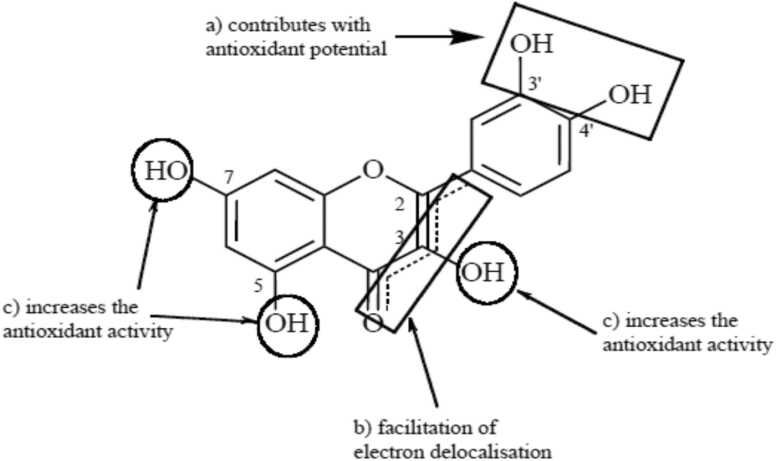
Summary of antioxidant structure-activity relationships of flavonoids.

Flavonoids are known to have high antioxidant activity. Many studies have shown significant differences in the antioxidant activity of the different flavonoid subgroups due to the many substitution patterns in their structures. Other studies discussed the structural effect on the antioxidant activity of flavonoids (Sichel et al., 1991; Rice-Evans et al., 1997). From these studies, the three main structural targets are summarized as follows (Fig. 6):

a) The 3'- and 4'-OH groups connected to the B-ring in an ortho position appear to stabilize their radical form. This site is believed to be responsible for metal chelation.
b) The 2, 3 double bond on the C-ring plays a decisive role in junction with the 4-oxo group and facilitates the electronic delocalization of the B-ring. In addition, the ketol structure of 4-keto and 3-OH or 5-OH appears to be another chelation site for metals.
c) OH groups attached to rings A and C at positions 3, 5, and 7 seem to increase the antioxidant capacity together with the 4-oxo groups.

### 3.4 Potential against inflammation

Inflammation is responsible for chronic systemic damage which can lead to many dangerous diseases. There is currently a growing understanding of the effects of diet on inflammatory diseases. Therefore, the effects of flavonoids as an essential part of a healthy diet have received more attention because of their anti-inflammatory effects [90].

**Fig. 7:**
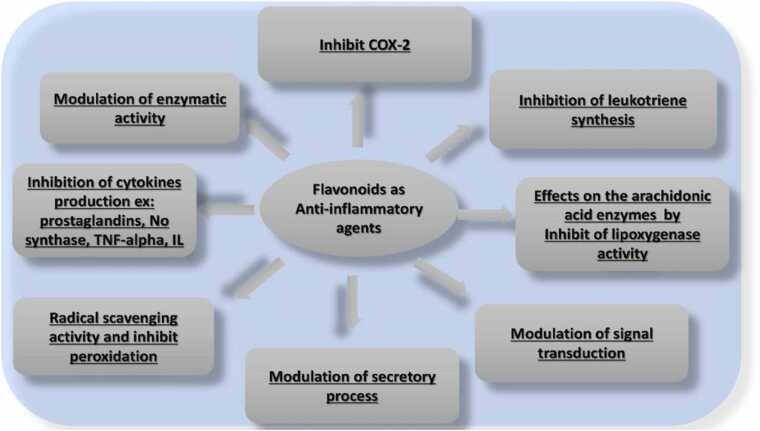
Flavonoid mechanism of anti-inflammatory activity.

Flavonoids exhibit pleiotropic effects and can modulate inflammatory regulatory nodes (Fig. 7). The anti-inflammatory effect of flavonoids can be mediated in many ways; a) antioxidant effects, b) inhibition of inflammation-related gene expression, c) interactions with signaling pathways, d) interactions with inflammation-inducing proteins.

#### 3.4.1 Anti-inflammatory mechanism of action

Flavonoids have anti-inflammatory activity through many actions including a) inhibition of transcription factors and regulatory enzymes that have a crucial role in the control of mediators involved in inflammation, b) additionally they are able to scavenge ROS and to enhance immune mechanisms and cells, c) modulation of secretory process, d) their effect on the arachidonic acid enzymes by inhibiting of lipoxygenase activity, e) modulation of signal transduction, f) inhibition of leukotriene synthesis, g) inhibition of cytokines production (Prostaglandins, No synthase, IL, TNF-alpha), h) modulation of enzymatic activity, i) inhibit COX-2 (Fig. 7). (Table 4).

**Table 4:** List of flavonoids with anti-inflammatory effect and their mechanism of action.

**Flavonoids**	**Mechanism of action**	**Reference**
Quercetin	Suppression of IgE	[208]
	Reduction of histamine	[27]
	Reduction in oxidative stress	
Kaempferol	Inhibit chemokines production	[62]
Baicalein	Activation of regulatory T cells	[22]
		[300]
Chrysin	Inhibit platelet function	[222]
Ruthenium-conjugated chrysin	Inhibit thrombus formation and platelets function	[221]
Genistein	Inhibit Pro-inflammatory cytokines	[127]
Puerarin	Decrease in inflammatory responses	[115]
	Decrease NF-kB activity	
Isoflavone	Suppress CD83 and CD80 expression	[170]
Epicatechin	Anti-allergic effects	[244]
Cyanidin	Attenuate inflammation in T cell	[154]
Anthocyanidin	Decrease adhesion between monocyte and endothelial cells	[61]
Luteolin	Decrease of prostaglandins and histamine release	[130]

#### 3.4.2 Structure activity relationship for anti-inflammatory

**Fig. 8:**
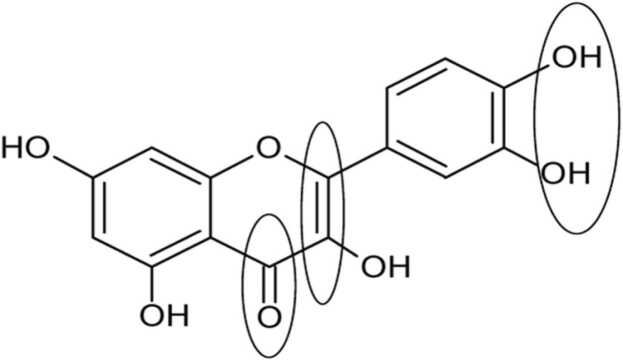
Summary of anti-inflammatory structure-activity relationships of flavonoids.

Typically, the structural activity of flavonoids as anti-inflammatory agents is examined as follows: a) -C = O groups at C-4 b) position and number of OH groups c) non-glycosylated d) methoxylated e) glycosides with high lipophilicity f) and ring unsaturation [91] (Fig. 8, Table 5).

**Table 5:** Summary of anti-inflammatory structure-activity relationships of flavonoids.

**Responsible structural**	**Mechanism of action**	**References**
2,3-double bond	Inhibit phospholipase A_2_	[128]
2,3-double bond	Inhibit COX-1	[128]
3′,4′-OH groups	Inhibition of inflammation-related gene expression	[53]
4-(-C = O) group		
2,3-double bond		
5,7-OH groups		
3-OH group	Inhibit lipoxygenase	[128]
2,3-double bond		
3-isoprenyl residue	Inhibit COX-2	[128]
2,3-double bond		
Galloyl moiety		
5,7-OH groups	Anti-inflammatory action	[53]
3′,4′-OH or OCH3 groups		
2,3-double bond		

The most important sites in flavonoids as anti-inflammatory are the C_2_ and C_3_ double bonds, 3 ', 4' OH in the B-ring and 5, 7 OH in ring A. The OH group is important for anti-inflammatory activity and for the inhibition of lipoxygenase activity [126]. Therefore, flavonols are more potent than flavones. The increase in the number of OH groups in ring B leads to increased anti-inflammatory activity. The introduction of the sugar fraction at the C_3_, C_7_ or C_8_ positions significantly reduce the anti-inflammatory activity, indicating the importance of structural lipophilicity and bioavailability [137]. In addition, the OH groups at C_4_ ', C_5_ or C_7_ and their arrangement are responsible for the activity. The C_5_ OH in A ring is important for activity because of its interaction with C_4_ carbonyl group (-C = O), which forms intramolecular hydrogen bonds and increases its activity, whereas substitution causes decreased activity. Likewise, the C_3_ and C_7_ OH groups are important for increasing activity, and their replacement decreases activity. The introduction of substituents at C_8_ leads to a slight decrease in activity [138]. The presence of the OCH_3_ group increases the inhibition of lipoxygenase activity because it increases the lipophilicity and bioavailability of flavonoids and changes the pharmacokinetic behavior [126].

### 3.5 Hepatoprotective activity

Flavonoids have apparently hepatoprotective effects (Tapas et al., 2008; ElGengaihi et al., 2016a, 2016b; Mossa et al., 2016) by inhibiting oxidative stress with increasing superoxide dismutase (SOD), catalase (CAT), and reducing malondialdehyde (MDA), nitric oxide synthase (iNOS). They reduce the levels of aspartate and alanine aminotransferase (AST and ALT, respectively) and pro-inflammatory cytokines in the serum and prevent the phosphorylation of NF-κB/p65, IKK, and IκBα in the NF-κB signaling pathway. Besides,flavonoids can inhibit hepatocyte apoptosis through suppressing caspase proteins and increasing Bcl-2 / Bax ratio [88]. Treatment with cyanidin-3-O-β-glucoside inhibits the release of inflammatory cytokines, reduces liver peroxidation, and prevents the development of hepatic steatosis (Zhu et al., 2012).

#### 3.5.1 Hepatoprotective mechanism of action

**Fig. 9:**
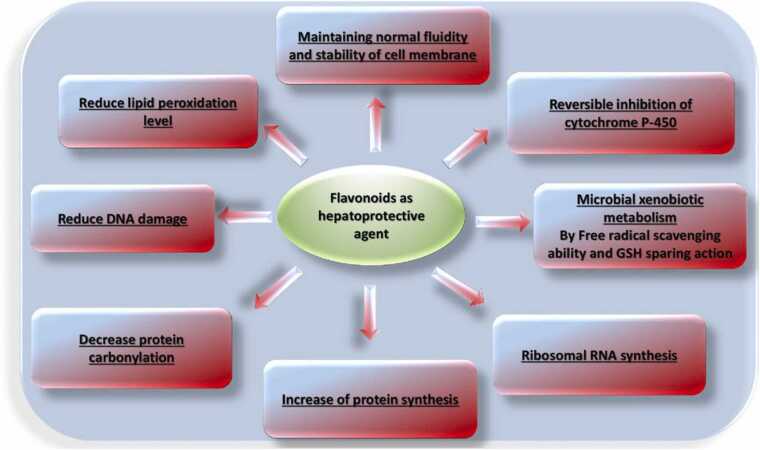
Flavonoid mechanism of hepatoprotective activity.

Flavonoids have hepatoprotective activity through many actions like maintaining normal fluidity and stability of cell membrane, reversible inhibition of cytochrome P-450, ribosomal RNA synthesis, reduction of lipid peroxidation level, reduction of DNA damage, and decrease of protein carbonylation (ElGengaihi et al., 2016b) (Fig. 9, Table 6).

**Table 6:** List of flavonoids with hepatoprotective effect and their mechanism of action.

**Flavonoids**	**Mechanism of action**	**References**
Apigenin	Inhibit Nrf2-signaling	Tsaroucha et al., 2016
	Activate BCL-2 apoptotic pathway	
**Catechin**	Modulate the expression of hepatic carcinoma factor	Yang et al., 2017
	Increase expressions of vital antioxidative signals	
**Curcumin**	Suppress cytokines, lipid peroxidation, hepatic stellate cells, and Akt activation.	Nabavi et al., 2014
	Induce expression of Nrf2, SOD, CAT, and GSH.	
**Epicatechin**	Downregulate liver enzymes	Shanmugam et al., 2017
Wogonoside	Increase oxidation process.	Wang et al., 2015
Resveratrol	Regulation lipogenesis.	[297]
	Reduce transcriptional factors, liver enzymesand cytokines.	
**Naringenin**	Upregulate Nrf2 pathways	Esmaeili and Alilou, 2014
	Increase CAT, SOD	
	Decrease AST, ALT, AP, GGT	
**Morin**	Suppress NF-Kβ signaling	Caselli et al., 2016
**Hyperoside**	Regulate detoxifying enzymes phase II	Xie et al., 2016
	Activate Nrf2 signaling pathway	Zou et al., 2017

It has been reported that silymarin increases the enzymatic activity of DNA-dependent RNA polymerase 1 and subsequently RNA, DNA and protein biosynthesis, that leads to cell proliferation, leading to regeneration of liver cells (Sonnenbichler et al., 1986). The therapeutic properties of silymarin include scavenging of ROS, collagen production, regulation of cell membrane integrity and permeability, inhibition of NF-κB activity, and inhibition of leukotrienes and kinase depression (He et al., 2004).

#### 3.5.2 Structure activity relationship for hepatoprotective activity

**Fig. 10:**
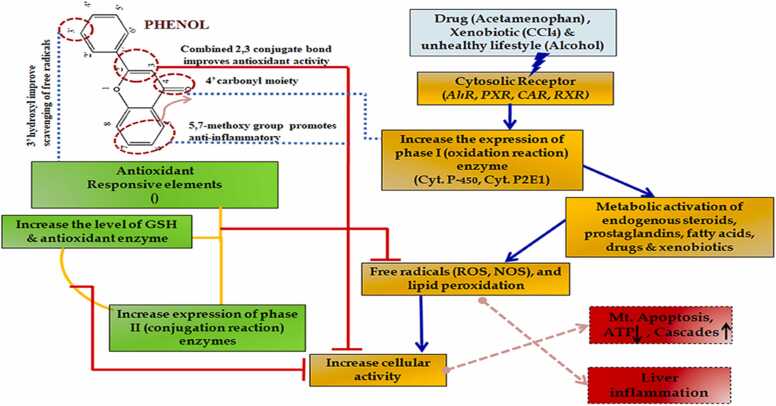
Summary of hepatoprotective structure-activity relationships of flavonoids.

The double bond at the C_2_ and C_3_ in ring A and the OH groups of C_3'_ or C_4'_ in ring B increases the protective activity, but the hydroxymethylation effect at C_3'_ and C_4'_ is reversed (Fig. 10). In addition, apigenin has good hepatoprotective activity and good potential as promising therapeutic anti-inflammatory agent [88].

### 3.6 Potential against hypertension

Mechanically, flavonoids mediate antihypertensive effects [230] by increasing the bioavailability of NO, modulating vascular ion channel activity and decreasing oxidative stress in endothelial cells. At the endothelial level, flavonoids exert a vasorelaxant effect mainly by elevating NO levels through various mechanisms such as increasing the bioavailability of NO, increasing eNOS activation via the PI3K / Akt / eNOS cascade and increasing Ca levels.

#### 3.6.1 Antihypertensive mechanism of action

**Fig. 11:**
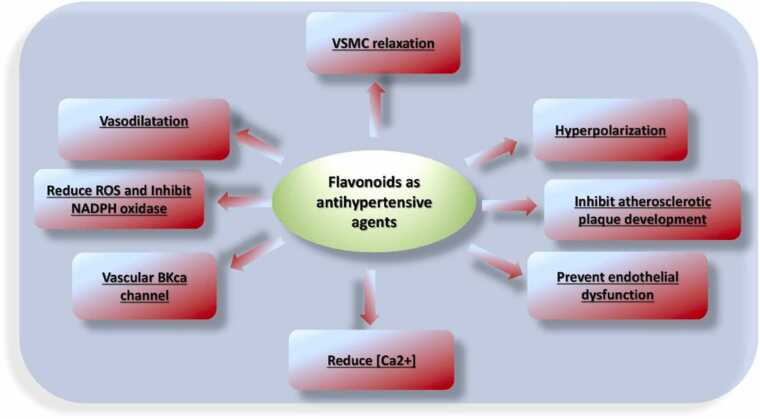
Flavonoid mechanism of antihypertensive activity.

Mechanistically, antihypertensive effect of flavonoids is mediated by increasing NO bioavailability, modulation of vascular ion channel activity or reduction of oxidative stress in endothelial cells (Fig. 11, Table 7).

**Table 7:** List of flavonoids with antihypertensive effect and their mechanism of action.

Flavonoids	Mechanism of action	References
Epicatechin	Antioxidant, reduce ROS and NO	[131]
	Vasodilatation	
Kaempferol	VSMC relaxation, vasodilatation	[162]
	Vascular Ca channel	[94]
Quercetin	Hyperpolarization, VSMC relaxation, vasodilatation	[236]
Naringenin	VSMC relaxation, vasodilatation	[235]
	Vascular BKca channel	[162]
Daidzein	Inhibit NADPH oxidase, antioxidant, reduce NO Vasodilatation	[200]
Hesperetin	Prevent endothelial dysfunction	[196]
	Inhibit atherosclerotic plaque development	[252]
	Increase NO generation	[155], [156]
	Reduce [Ca2 + ]	[248]

#### 3.6.2 Structure activity relationship

**Fig. 12:**
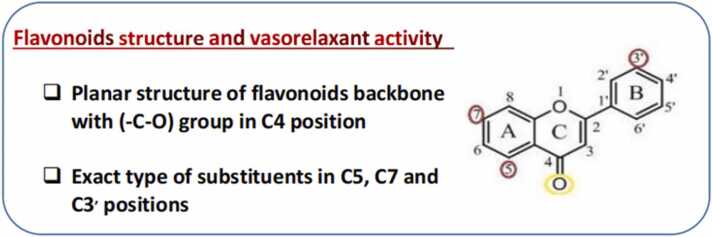
Summary of antihypertensive structure-activity relationships of flavonoids.

In general, there are two speculations that could be responsible for the high vasorelaxant effect of flavonoids: a) those with a planar structure, the same flavonoid basic skeleton and the -C = O group attached to the C_4_ position of the C ring, b) those with the same substituent attached to the C_5_ position of A ring and the C_3'_ and C_4'_ positions of ring B (Fig. 12).

### 3.7 Potential against cardiovascular disease

Currently, flavonoids are attracting a lot of attention in the prevention of cardiovascular diseases (CVD). Foods rich in flavonoids have a positive effect on CVD. Evidence for the activity of metabolized and unmetabolized flavonoids in the three defense pathways in heart diseases is highlighted: NO bioavailability, induction of antioxidant enzymes, and anti-inflammatory processes.

#### 3.7.1 Cardioprotective mechanism of action

**Fig. 13:**
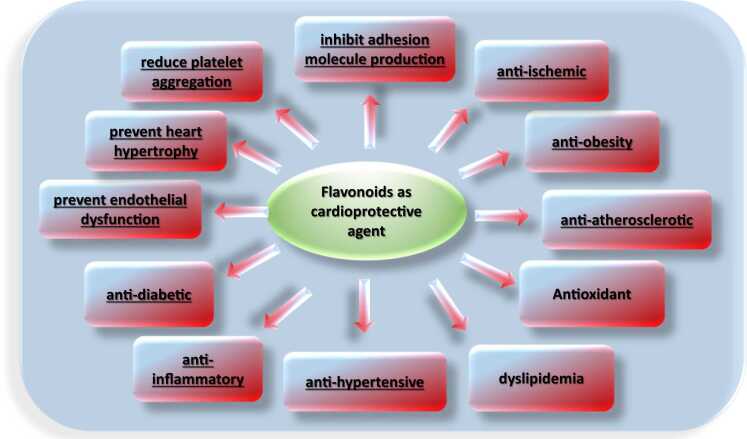
Flavonoid mechanism of cardioprotective activity.

Flavonoids have a positive effect on the cardiovascular system through various mechanisms. Although the direct mechanism is not understood, the effects of flavonoids appear to be diverse and dependent on many processes. The main pathways include anti-inflammatory and antioxidant activity, anti-platelet effect, anti-ischemic, anti-obesity, anti-atherolsclerosis, dyslipidemia, anti-hypertensive, anti-diabetic, prevent endothelial dysfunction, prevent heart hypertrophy, inhibit adhesion molecule production, regulating blood pressure, lowering cholesterol, and protecting LDL from oxidation (Fig. 13, Table 8). Flavonoids can reduce the inflammatory process via a variety of mechanisms, including NO inactivation, and inhibition of the entry of leukocytes into inflammatory sites [166]. In addition, flavonoids improve vascular function and modulate vascular endothelial inflammation [82]. Besides, flavonoids decrease the activity of enzymes that produce ROS, lipoxygenase, NADPH oxidase, and xanthine oxidase [165]. Flavonoids increase adenosine monophosphate kinase activity leading to inhibition of the rate-limiting enzyme for cholesterol synthesis [268]. Inhibition of COX and lipoxygenase by flavonoids leads to reduction in thromboxane and leukotriene synthesis and thereby leads to decrease in vasoconstriction [98]. Flavonoids showed decreased vascular cell adhesion molecules and C-reactive protein [163]. Flavonoids' inhibitory action of platelet aggregation is associated with the inhibition of the compounds that impair endothelial function and the formation of NO in the vascular endothelium [260].

**Table 8:** List of flavonoids cardioprotective effect and their mechanism of action.

Flavonoids	Mechanism of action	References
Cyanidin	Increase eNOS	Xu et al., 2007
	Increase Thioredoxin	
Quercetin	Increase eNOS activity	Shen et al., 2012
	Increase Phosphorylation of eNOS	
	Decrease HOCl‐induced endothelial dysfunction	
Proanthocyanidin	Increase NO production	Qian et al., 2017
Resveratrol	Increase eNOS	
Cyanidin‐3–glucoside	Increase eNOS	Edwards, et al., 2015
Luteolin	Enhance relative coronary flow	[24]
	Induce vasorelaxion	[117]
	Reducing oxidative stress	[31]
	Prevent ischemia-reperfusion injury	
	Regulate potassium and calcium channels	

#### 3.7.2 Structure activity relationship for cardioprotective activity

The sequence of effectiveness of cardioprotective flavonoids is as follows descendingly; apigenin and luteolin, and kaempferol and quercetin followed by genistein and daidzein, then naringenin, then floretin and finally catechins then epicatechins. Analysis of the relationship between structural activities revealed that 5-OH, 7-OH, 4'-OH are essential for good cardioprotective activity. While, the presence of a glycosylated group significantly reduces cardioprotective activity. In addition, molecular volume and total energy predict the cardioprotective activity of flavonoids.

### 3.8 Potential against ulcers

Flavonoids are one of the most important types of phytocompounds used in ulcer therapy especially to combat *Helicobacter pylori* (*H. pylori*) [5]. Rutin was investigated for its anti-ulcer effect against gastric lesions due to its anti-lipoperoxidation effect in addition to its antioxidant potential, which reduces gastric MPO activity, increases nitrite / nitrate, exhibits NO production and increases GSH activity [83]. The various flavonoids of *Oroxylum indicum* have been used for centuries to treat various gastric ailments [249]. It was also found that several substituted flavones showed good gastroprotective activity. Flavonoid glycosides exhibit gastroprotective properties in mice exposed to multiple ulcer causes. It has been demonstrated that 5-methoxy-49-fluoroflavone is very effective as anti-ulcer agent [16].

#### 3.8.1 Antiulcer mechanism of action

**Fig. 14:**
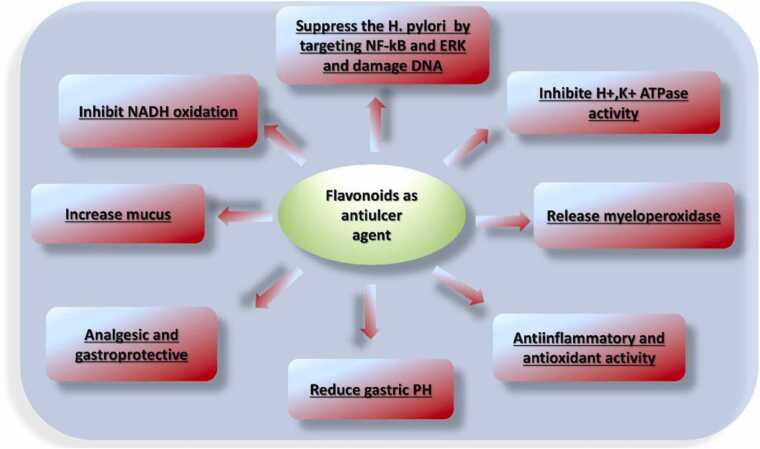
Flavonoid mechanism of gastroprotective activity.

Flavonoids provide a cytoprotective effect by increasing levels of endogenous prostaglandins, increase mucus, reduce gastric PH, release myeloperoxidase reducing histamine secretion, inhibiting *H. pylori*, scavenging ROS and antisecretory mechanisms (Fig. 14, Table 9) [191], [51]. The gastroprotective effect of resveratrol is sufficiently based on its potential to inhibit the production of important inflammatory mediators, to inhibit the expression of NF-κB and intracellular transcription enzymes (MAPKs) [110] and to decrease gastric MPO activity, decrease MDA, increase the collagen content and restore depleted GSH. Flavonoids play an important role in its therapeutic function in gastric tissue by inhibiting TNF-α. These polyphenols also reduce the elevated levels of lucigenin and luminol chemiluminescence, which indicate a significant inhibition of intracellular and extracellular oxidative events in the gastric mucosa.

**Table 9:** List of flavonoids with gastroprotective effect and their mechanism of action.

Flavonoids	Mechanism of action	Ref
Flavones and flavonols	Inhibit H. pylori	[164]
Artemisin	Bactericidal kinetics	[42]
	Morphological degeneration	
Pinostrobin \	Decrease gastric motility	[2]
Catechin	Urease inhibitor	[171]
	Anti-inflammatory	[251]
		[226]
Isorhamnetin	Inhibit ulcer	[289]
	Eradicate H.pylori	[259]
Curcumin	Inhibit proton potassium ATPase	[294]
	Chemo-preventative	[112]
4-methoxy quercetin-7-O-glucoside	Chemopreventive	[220]
		[103]
Glabridin	Anti-adhesive activity	[17], [279]
	Inhibit dihydrofolate reductase	
	Inhibit DNA gyrase	
licoricidin	Chemopreventive agents	[71]
		[11]
Leucocyanidin	Increase mucus	[145]
		[113]
Cabreuvin	Inhibit NADH oxidation	[190]
Baicalein and chrysin,	Gastroprotective	[15]
		[249]
Vitexin	Release myeloperoxidase Inhibite H+ ,K+ ATPase activity *N*-	[215]
	Acetylation	
Quercetin	Anti-inflammatory	Wang et al., 2015
	Antiulcer invivo	
	Analgesic	
Emodin	Damage DNA *H. Pylori*	[271]
Kampferol	Reduce gastric PH	[169]
	Participate No and SH	
Rutin	ulcer-protecting effects against gastric lesions	[136]
Resveratrol	Chemopreventative	[204]
	Antioxidant	
7-carboxymethyloxy-39,49,5-trimethoxyflavone	suppresses the H. pylori–induced IBD by targeting NF-kB and ERK	[267]
		[109]

#### 3.8.2 Structure activity relationship

The presence of a OCH3 group at the position C-7 appears to enhance gastroprotection. The presence of OH groups in C7 and C5 in flavones reduces their gastroprotective activity. The double bonds in the intact C-2 and C-3 and C-ring appear to be required for the strong activity [180]. Replacing the aromatic B ring with eitheralkyl group or heterocyclic ring or indole does not alter the gastroprotective properties [30].

### 3.9 Potential against diabetes

Flavonoids, which have strong antioxidant activity, are believed to be beneficial for treating diabetes [100]. The potential of antioxidants to protect against harmful effects of hyperglycemia, as well as to improve the metabolism and absorption of glucose, should be viewed as a major alternative in diabetes treatment [181]. In addition to their antioxidant effects, flavonoids can act on α-glycosidase which is considered as one of the biological targets involved in diabetes type 2. As free radical scavengers, flavonoids can effectively prevent and / or treat diabetes type 2.

#### 3.9.1 Antidiabetes mechanism of action

**Fig. 15:**
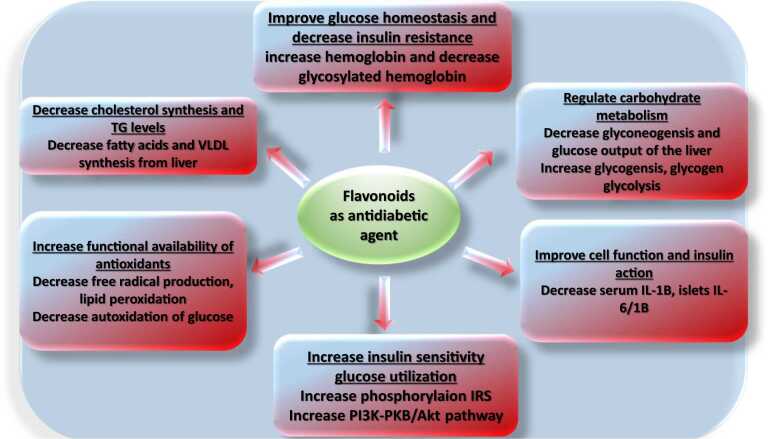
Flavonoid mechanism of antidiabetic activity.

Flavonoids have a beneficial effect on diabetes through many pathways such as a) decrease cholesterol synthesis and TG levels, increase functional availability of antioxidants, increase insulin sensitivity glucose utilization, improve cell function and insulin action, reduce carbohydrate metabolism (Fig. 15), they interact with various signaling and metabolic pathways in pancreatic β cells, skeletal muscle, adipose tissue, and liver. Flavonoids increase glucose absorption by white adipose tissue and skeletal muscle. They affect β cell function, mass, insulin sensitivity, energy metabolism and stimulate protein kinases, which are essential for maximum glucose uptake stimulation [21].

#### 3.9.2 Structure activity relationship for Anti-diabetes

A study Xu (2010) reported that the di-OH groups at the C_3'_and C_4'_ positions were effectively conjugated to α-glucosidase.

The lack of C_2_-C_3_ double bonds and ketone groups on C_4_ in the C ring reduces the inhibitory activity of α-glucosidase and xanthine oxidase. In addition, the presence of a cathecholic system in B ring in the absence of the C_2_,C_3_ double bond and the ketone group at the C_4_ position is not significant enough to demonstrate antidiabetic effects. In addition, the acetylation or alkylation of the OH groups in ring A decreases flavonoids bioactivity, demonstrating their inability to interact with enzyme binding sites and scavenging ROS.

**Fig. 16:**
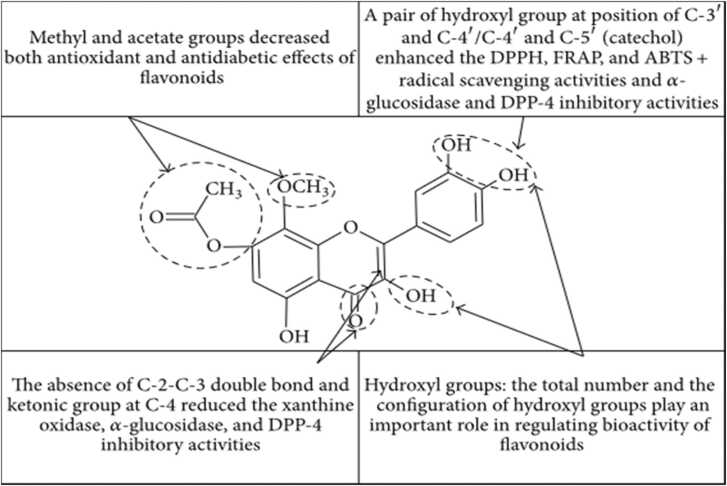
Summary of antioxidant structure-activity relationships of flavonoids.

In summary, the results of the antidiabetic analysis indicate that the chemical criteria for the flavonoids bioactivity are very important (Fig. 16). The alkyl substitution is important determinant of antidiabetic activity when compared to spine alone. Both the configuration and the number of OH groups have a significant influence on the radical scavenging mechanism [253] and the antidiabetic effect. Therefore, the hydroxyl-configuration, number of OH groups, C_2_,C_3_ double bonds and functional C_4_ ketone groups are the main structure features of flavonoid bioactivities, especially with regard to the antidiabetic effect.

### 3.10 Potential against fungal infections

Fungal infections cause high mortality rates worldwide. The incidence of increasing drug resistance in fungal diseases continues to increase. The scenario for the existing antifungal drugs and their complications is critical. Antifungal drugs have limitations: high toxicity, renal failure, and low performance. Therefore, it is important to seek new treatments, such as alternative therapies, that may be more active against most fungal diseases. Plants and herbs that contain flavonoids are known for their many therapeutic activities. Various flavonoids have been studied for their antifungal activity and are perhaps the promising, and most potent agents for inhibiting fungal infection [104], [12], [197], [231]. They often inhibit fungal growth in various mechanisms of actions and increase plasma membrane damage and mitochondrial dysfunction, and inhibit cell wall formation, cell division, protein synthesis and the pumping system. These flavonoids are capable and effective in synergistic combination therapy with conventional drugs, which may be more suitable and supportive in finding new drug therapies to fight fungal pathogens ([205]; Jin, Y.S., 2019).

#### 3.10.1 Antifungal mechanism of action

**Fig. 17:**
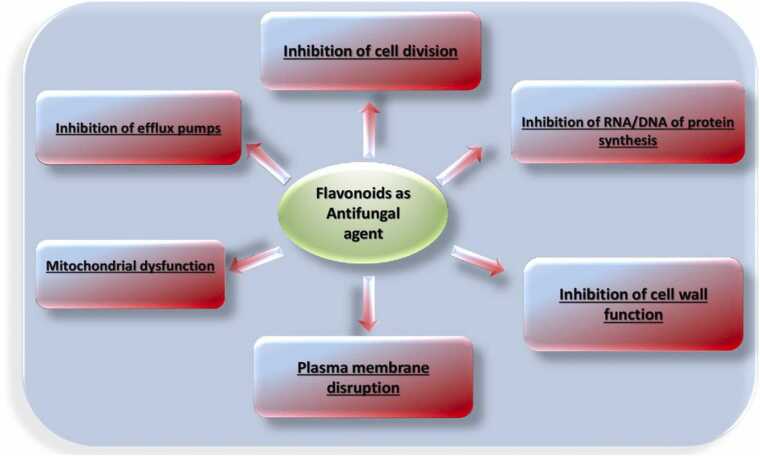
Flavonoid mechanism of antifungal activity.

Flavonoids have been widely used for centuries to inhibit fungal growth through various mechanisms (Fig. 17, Table 10). The way flavonoids work as antifungal agents is based on the induction of apoptosis, DNA fragmentation, mitochondrial damage, accumulation of ROS, etc.

**Table 10:** List of flavonoids with antifungal effect and their mechanism of action.

Flavonoids	Mechanism of action	References
Baicalein	Disrupt plasma membrane induce apoptosis Elevates ROS	[120] [241] Tsang et al., 2010
Catechin	Activate phosphatidylserine Inhibits fatty acid synthase Increase ROS Induce apoptosis Mitochondrial depolarization DNA fragmentation	[57]
Glabridin	Decrease cell size Increase membrane permeability DNA fragmentation Chromatin condensation	[179]
Wogonin	Accumulate ROS in mitochondria Decrease membrane potential Reduce ATP synthesis	[58]
Resveratrol, curcumin and quercetin	Inhibit oxidative phosphorylation t Increase ROS in mitochondria Modulate transcription factors activity Control mitochondrial proteins’ expression Exhibit proapoptotic functions Upregulate Bcl-2 expressions Downregulate anti-apoptotic proteins	[192], [193] [73] [78]
Apigenin	Disrupt plasma membrane Inhibit cell cycle	[142]
Chrysazin Alizarin	Suppress biofilm formation Inhibit hyphal formation Inhibit the cell cycle	[167]
Honokiol Magnolol	Inhibit effects on the cell cycle and biofilm-formation	[250]
Daphnegiravone D	Inhibit cell division Arrest G0/G1 phase Induce apoptosis Reduce CDK2, CDK4 and cyclin E1, expression Increase caspase 3 and PARP	[270]
Baicalein	Inhibit lipooxygenase Inhibit efflux pump	[97]
diorcinol D	Inhibit efflux pump decrease Cdr1 expression	[148]
Apigenin, luteolin, wogonin, tangeritin, baicalein scutellarein, chrysin,	Inhibit efflux pumps Induce cell death	[293] [241] [238]
sedonan A	Inhibit efflux pumps Disturb various intracellular transcription	[29]
Dorsmanin	Inhibit efflux pumps	[174]
5-flurocytosine	Inhibit nucleic acid synthesis formation of fluorinated pyrimidine metabolites, deficit of cytosine deaminase Deregulate pyrimidine biosynthesis	[185]
Catechin	Inhibit nucleic acid synthesis Reduce the hypha-specific gene expression Inhibit FCS-induced hyphal formation	[229]
Myricetin, kaempferol, fisetin, luteolin naringenin genistein	Inhibit filamentous fungus Cochliobolus lunatus Inhibit nucleic acid synthesis	[40]
Apigenin	Interfere with the translational activity of fungal foot-and-mouth disease	[213]
Carvacrol	Inhibit nucleic acid synthesis Disrupt the cellular cytoplasmic membrane Induce apoptosis	[305]
Lico A	Biofilm formation Inhibit glucan synthase, ergasterol synthesis and efflux pumps Induce apoptosis Disrupt cell wall	[37]
Fisetin	Inhibit ergasterol biosynthesis	[223]
Isoquercetin	Bind to ergosterol and disrupt cell membrane	[129]
Baicalein	Biofilm formation	[38] [135]
Glabridin	Inhibit nucleic acid synthesis	[44]
Apigenin	Inhibit glyoxylase cycle Induce cell shrinkage	[142]
Silymarine	Disrupt membrane Increase membrane permeability Decrease membrane fluidity Membrane depolarization and K+ leakage	Yun and Lee 2018

#### 3.10.2 Structure activity relationship for antifungal activity

**Fig. 18:**
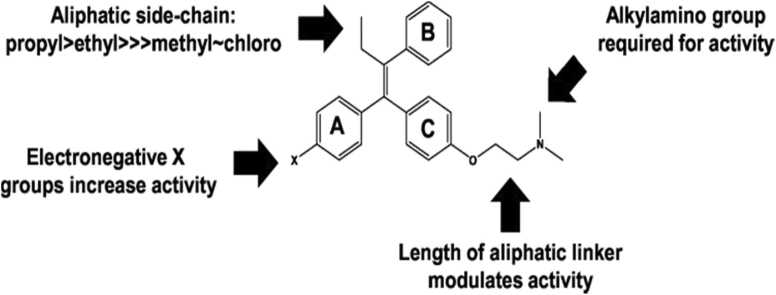
Summary of structure-activity relationships of taxifolin as antifungal agents.

The three main molecular properties that affect the antifungal activity (Fig. 18) are as follows:

a) Electronegative aromatic ring substituents that moderately increase the activity
b) The presence of an alkylamino group attached to one of the aromatic rings of the triphenylethylene core
c) A suitably sized aliphatic substituent at position 2 of ethylene group.

### 3.11 Potential against cancer

Cancer is a terrible disease all over the world and one of the biggest problems for human health. New techniques are needed for successful treatment. Many limitations have been noted with conventional treatments, including the high cost and high toxicity of current cancer drugs. Such a situation poses great challenges for all scientists and requires the development of new drugs that are environmentally friendly and have a more financially sound methodology. In this context, the high biodegradability and biocompatibility of phytocombinants increase their effectiveness in treating cancer [1]. In this sense, special attention is paid to improve cancer drugs using plant phytocompounds. Their potential, availability and low cost compared to modern therapeutic drugs for the treatment of dangerous diseases make them more attractive [184] (El Gengaihi et al., 2016a, 2016b).

#### 3.11.1 Anticancer mechanism of action

**Fig. 19:**
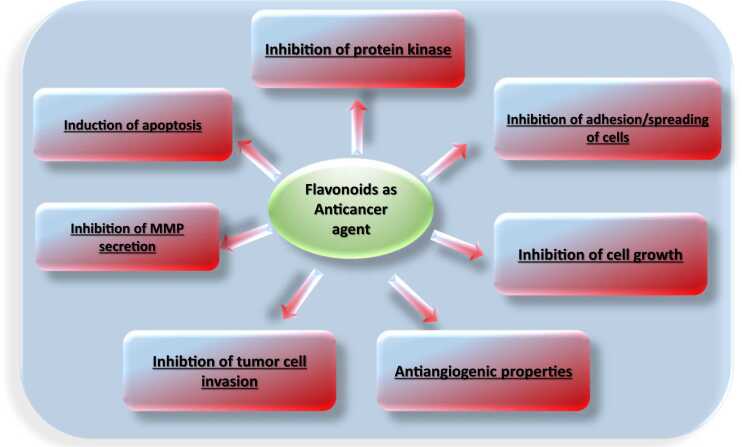
Flavonoid mechanism of anticancer activity.

So far, various mechanisms have highlighted the role of flavonoids in cancer therapy (Fig. 19, Table 11), including inhibition of proteasomes, induction of apoptosis, differentiation and cell cycle arrest [132], [133], [243], inhibition of nuclear factor signaling [13], and receptor interaction [96]. In addition, flavonoids may exhibit specific cytotoxicity for cancer cells, which is drawing much attention to flavonoid cytostatics as anticancer prodrugs [296].

**Table 11:** List of flavonoids with anticancer effect and their mechanism of action.

Flavonoids	Mode of action	References
Genistein	Increases expression of Bax, P2, GTP, glutathione peroxidase Inhibit topoisomerase II and NF-kB	[168] [160]
Apigenin	Caspases activation, GSH, GST, GPxn, GTP, STAT3 Inhibit signal transducer Block phosphorylation of JAK2 and STAT3	[240], [28]
Resveratrol	Increase p53 and Bcl-2 of X protein Decrease PI3K, Akt, MMP, Bcl2 Reduce MAP kinase phosphorylation Inhibit angiogenesis G1, G2, M phase arrest	[33] [202] [263]
Kaempferol	Activation caspase 3, p53 Cdc2, CDK2, CDK4, inhibition G1, G2, M phase arrest	[139] [80]
Chrysin	G1, G2, M phase arrest Induce apoptosis	[121] [228]
Flavopiridol	Inhibit cyclin dependent kinase Inhibit Topoisomerase-1 Inhibit COX-1	[266] [111]
Cyanidin	Inhibition of COX-1 and II MMP-2 and 9 ErK, JNK TNF alpha	[125]
Silamarin	Induce apoptotic factors Inhibition of anti-apoptotic factors G1, G2, M phase arrest	[140] [254]
Epigalloca Techingallate	Stimulate genes expression of tumor suppression	[183] [214]; Qiao et al., 2017;[206]
Oroxylin A flavone	Decrease COX-2 andi NOS Block NF-kB Block IkB degradation	[45] [81]
Quercetin	Scavenge ROS Cell proliferation signaling pathways NF-kB, MAPK, STAT3, PI3k/Akt, mTOR Decrease growth factors Induce apoptosis and cell cycle arrest	[23] [158]
Luteolin	Induce cell cycle arrest Induce apoptosis Cytoskeleton shrinkage	[106]

#### 3.11.2 Structure activity relationship for anticancer activity

**Fig. 20:**
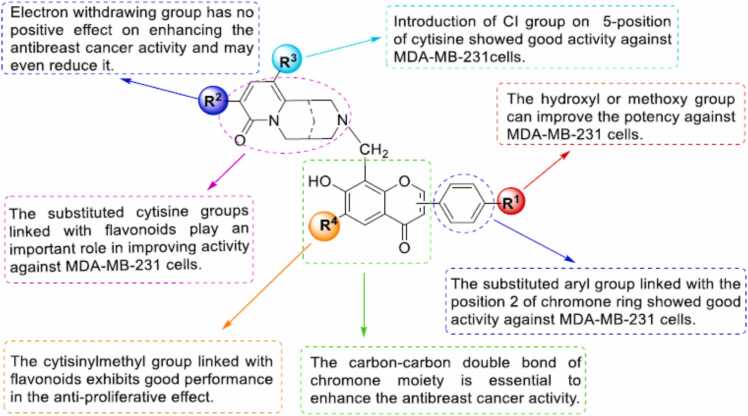
Structure activity relationship of cytisine-flavonoid conjugates as potent anti-breast cancer agent.

The important role of the C_2_ =C_3_ double bond is essential for strong tumor inhibition [132], [133], [96]. In addition, greater inhibition will occur if the two hydroxyl groups of ring B exist side by side and C_2_ =C_3_ is unsaturated [96]. It should be noted that many reports provide evidence of the effect of hydroxylation on tumor modulation. Specific hydroxylated flavonoids have a stronger inhibitory effect on cancer cells than permethoxylation analogs. It is proposed to replace the B ring as a catechol part with vital influence. Meanwhile, the additional substitution of hydroxyl groups on ring B does not change the activity [132], [133]. In the case of the C ring, 3-hydroxylation is seen as a very important component in enhancing the biological effect [13]. The flavonoid derivatives of O-methylation contribute to increased biological activity, which is often associated with ring A polymethoxylation. According to previous studies, glycosylation does not contribute to the induction of cell differentiation [132], [133] (Fig. 20).

### 3.12 Potential against bacterial infection

The development of antibiotic resistance in bacteria is a global problem that requires the search for more potent phytocompounds derived from nature to overcome this problem. Flavonoids are phytocompositions with antibacterial, antioxidant and anti-inflammatory potential. In this way, flavonoids can be developed into new antimicrobial agents in food and therapeutical products.

#### 3.12.1 Antibacterial mechanism of action

**Fig. 21:**
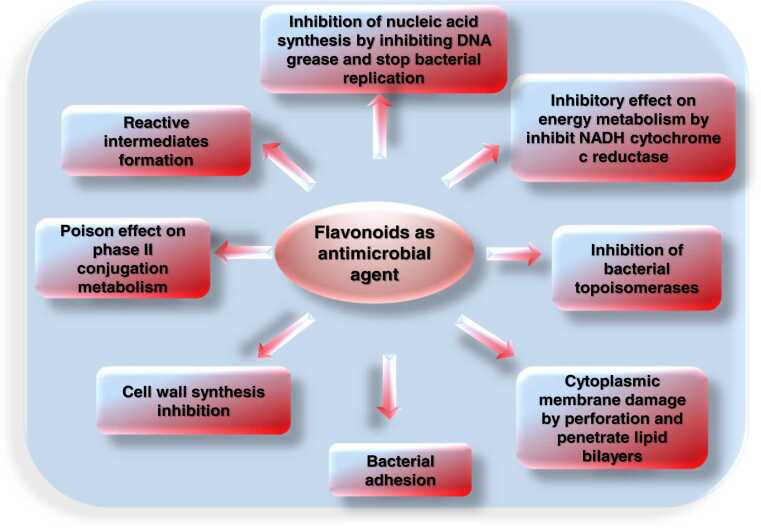
Different actions of flavonoid on bacterial cells.

The proposed flavonoid antibacterial mechanisms (Fig. 21, Table 12) are mainly as follows: Inhibition of energy metabolism, inhibition of cell proliferation, inhibition of nucleic acid synthesis, reduction of biofilm formation and cell adhesion, attenuation of pathogenicity [54] and damage to membranes possibly by producing hydrogen peroxide (Cushnie and Lamb, 2005).

**Table 12:** List of flavonoids with antimicrobial effect and their mechanism of action.

Flavonoids	Mode of action	References
Silymarin	Inhibit ATP synthase	[75]
Chalcon	Inhibit NADH-cytochrome c reductase activity	[86]
Quercetin	Inhibit refflux pumps Decrease lipid peroxide Inhibit DNA gyrase and protein kinase Disrupt cell membrane	[46] [242] [257]
Apigenin	Inhibit peptidoglycan crosslinking Inhibit dehydratase and protein kinas	[242]
Naringenin	Disrupt membrane Inhibit nucleic acid synthesis	[64]
Epicatechin	Inhibit dihydrofolate reductase Inhibit quorum sensing	Cushnie et al., 2011
Myricetin	Inhibit helicase	[239]
Luteolin	Inhibit topoisomerase	[272] [79]
Kaempferol	Inhibit bacterial virulence	[176]
Taxifolin	Inhibit peptidoglycan synthesis and fatty acid synthase	[76]
Glabridin	Inhibit DNA gyrase and dihydrofolate reductase	[17]
Emodin	DNA damage	[63]
Catechin	Disrupt cell membrane Damage cytoplasmic membrane by perforation	[217]

#### 3.12.2 Structure activity relationship for antibacterial activity

**Fig. 22:**
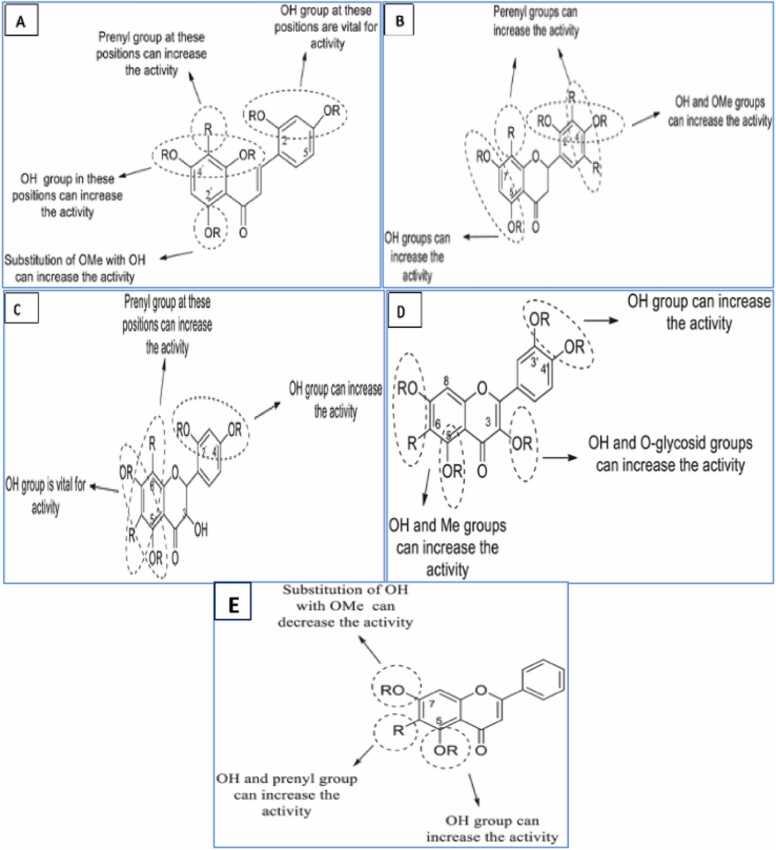
Summary of antibacterial structure-activity relationships of A) chalcones, B) flavans, C) flavanols, D) flavonols and E) flavones.

The amphipathic properties of flavonoids play an important role in their antibacterial properties [65]. Hydrophobic substituents like alkyl chains, alkylamino chains, prenyl groups and heterocyclic units containing oxygen or nitrogen usually increase flavonoids antibacterial activity [285]. The number and position of the prenyl groups in ring A increased activity, but the addition of the prenyl groups to another ring decreased activity. In addition, it has been reported that the presence of OH groups at different positions on rings A and B increases antibacterial activity [172], [173], [194], [195]. The number of glycosyl groups instead of OH groups at position 3 also plays a crucial role in the antibacterial activity (Fig. 22). The only substitution that reduces activity is methoxylation of position 3 [20].

### 3.13 Potential against viral infection

Viral infections are very difficult to control than bacterial infections, while antiviral agents are the least available. Natural phytocompounds provide a powerful resource for antiviral agents. Flavonoids exhibited potent antiviral activity (Table 13) [295]. Flavonoids stop HIV cell by the phosphorylation of proteins and inhibition of cytokines [147], [150], [19], [201].

**Table 13:** Antiviral potentialities of some flavonoids and their mechanism of action.

Flavonoid	Activity against virus	References
Glabranine 7-O-methyl-glabranine	Dengue virus	[280]
5-hydroxy-7,8-Dimethoxyflavone	Anti-influenza viruses	Wu et al., 2010
Vitexin	Para influenza type3 virus	Peterson 1991
Orientin	Para influenza type3 virus	Pang et al. 2013
Quercetin	HCV, polio, herpes simplex	Chwil et al. 2014
Naringenin	HCV	Ashfaq and Idrees 2014 Nahmias et al. 2008
Apigenin	Anti-influenza viruses, HCV, Enteovirus-71	Grienke et al. 2012 [296]
Quercetin	Mayaro virus	Santos et al. 2014
7-hydroxyisoflavone	Enterovirus71	Wang et al. 2013
Acacetin	Anti-influenza viruses	Wu, Yu et al. 2010
Liquiritigenin	HCV	Adianti et al. 2014
Chrysosplenol C Pterocaulonsphacelatum	Polio virus	Bhatty 1999 Rocha Martins et al. 2011
Eudraflavone B hydroperoxide	Herpes simplex type 1 virus	Du et al. 2003
Moralbanone	Herpes simplex type 1 virus	Farmer et al., 2012
Ladanein	HCV	Haid et al. 2012
Leachianone G	Herpes simplex type 1	Zafar et al. 2013
Baicalin	HIV	[147]
Myricetin	HIV	[201]
Flavonol-7-O-glucoside herbacitrin	HIV-1	[19]

#### 3.13.1 Flavonoids potentiality against CoVs

**Fig. 23:**
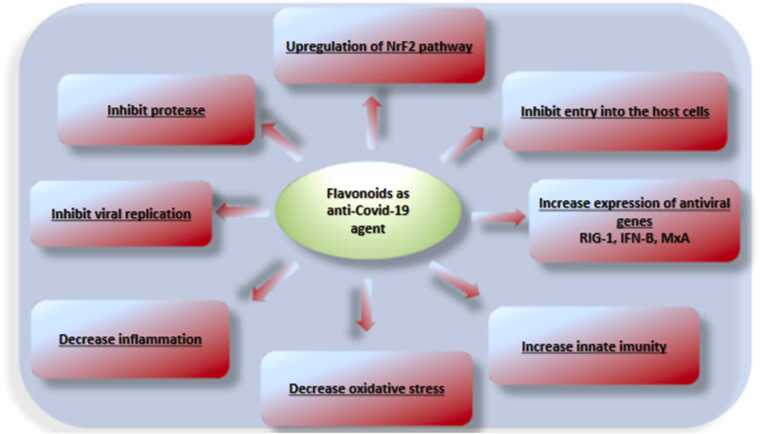
Different actions of flavonoid on CoV.

Coronavirus is responsible for the increasing severity of death causing COVID-19 disease. However, there is still a lack of antiviral drugs that are effective against the coronavirus. In short, there is a worldwide need for concerted efforts to combat such disease in the future. Most of the publications focus on polar compounds. Compounds that show promise in inhibiting coronavirus are scotelarein, silivestrol, tryptanthrin, saicozaponin B2, myricitin, quercetin, caffeic acid, isabavacalcone, and psoralidin. The most promising small molecule identified as a coronavirus inhibitor has been found to contain a conjugated fused ring structure, most of which are classified as flavonoids. An important area of research is the inhibitory effect of flavonoids on the coronavirus. Flavonoids existing naturally offer a large amount of biological diversity, including antiviral activity, and therefore may be useful as therapy against coronavirus infection. Flavonoids can prevent or modulate SARS-CoV-2 infection by many mechanisms (Fig. 23, Table 14) such as inhibiting spike glycoprotein, N protein, TMPRSS2 replication protein, ACE-2 entry receptor, protease, helicase, RNA-dependent RNA polymerase, activating Nrf2, and stimulating innate immunity ([295]; Antonio et al., 2020; Fuzimoto and Isidoro et al., 2020; [50], [227], [264], [265], [283]).

**Table 14:** List of flavonoids inhibiting corona virus and their mechanism of action.

Flavonoids	Mechanism of action	References
Quercetin	Inhibit viral replication Inhibit viral entry into the host cells Block interaction sites Stop viral spread	Jo et al., 2019 [264], [265]
Theaflavin-3,3-digallate	Inhibit protease Suppress viral replication	[47]
Resveratrol	Suppress viral replication by inhibiting N protein	[146]
Luteolin	Inhibit viral entry into the host cells	Yi et al., 2004
Bavachinin Neobavaisoflavone Isobavachalcone corylifol	Inhibit protease	[127]
Psoralidin	Inhibit protease	Ho et al., 2007
Juglanin	Blocks the 3a channel and inhibit virus release	Schwarz et al. 2014
Myricetin scutellarein	Inhibit helicase	Yu et al., 2012
Kampferol	Interact with coronavirus catalytic site	[119]
Emodin	Inhibit spike glycoprotein	[237]
Theaflavin	Inhibit RNA-dependent RNA polymerase (replication enzyme)	[159]
Hesperetin, hesperidin Naringin, naringenin	Inhibit ACE2, major receptor of corona virus	Cheng et al., 2020 [175]
Herbacetin, rhoifolin, pectolinarin	Inhibit protease by forming H bonds in the active site	Kim et al., 2020
5,7,3’,4’tetrahydroxy-2’-(3,3- dimethylallyl)isoflavone	Form H bond with protease receptors	[211], [212]
Quercetin-3 galactoside	Competitive inhibition of papain-like protease	[48]
Tomentin	Inhibit protease	[49]
Papyriflavonol A	Inhibit protease	[199]
Cyanidin	Inhibit RNA polymerase Halting viral replication	[264], [265]
Quercetin, phloretin, daidzein, arbutin, genistein, fisetin, myricetin, liquiritin, kaempferol, eriodictyol and chalconaringenin	Inhibit spike protein and therefore inhibit viral spread	[264], [265]
Naringenin	Inhibit 3CLpro Inhibit ACE2 receptor Inhibit replication	[258]

The sequence of effectiveness of anticovid-19 flavonoids is as follows kaempferol > quercetin > luteolin-7-glucoside > demethoxycurcumin > naringenin > apigenine-7-glucoside > oleuropein > curcumin > catechin > epigallocatechin > zingerol > gingerol > allicin [123].

#### 3.13.2 Structure activity relationship for antiviral activity

Structurally, the antiviral activity increases with the decrease in the number of OH groups in the B-ring. Meanwhile, the C_2_ =C_3_ double bond present in the C ring is seen as an important element which is beneficial for antiviral activity. In addition, trifloroside belongs to the group of dihydrocarbons without a flavonol structure, which has very little antiviral activity. This may be due to the hydrogen bonding formed by the galloyl group with amino acid residues at the active site of the enzyme [43].

Flavonoids exhibited significant binding at the N_3_-binding site compared to the main CoV protease inhibitor currently used, darunavir. The flavonol basic structure and the presence of a routine unit at position 3 in ring C and the absence of OCH_3_ group on the B ring of the flavonol structure can increase the anti-COVID-19 activity [295].

**Fig. 24:**
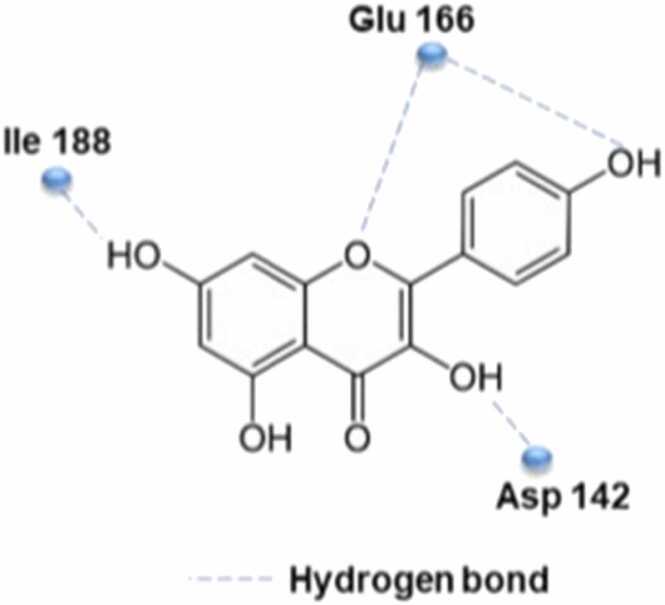
Interaction sites in kaempferol with CoV catalytic site by formation of hydrogen bond.

Fig. 24 shows the interaction between phenyl group in kaempferol and corona virus catalytic center, which is the hydrophilic task of the corona virus through hydrogen bonding with Glu166. Another hydrogen bond is formed between the OH group and Asp142, Ile188, while the chromen-4-one backbone is at the hydrophobic S2 site [119].

## 4 Conclusion and future approaches

In order to summarize the ongoing review, some main points are to be highlighted. Flavonoids could be effective drugs against the most dangerous degenerative diseases in the future. Compared to other natural plant phytochemicals, flavonoids can significantly enrich the pathways of breast cancer, Huntington's disease, Alzheimer's disease, insulin resistance, and drug resistance. In this regard, its versatile therapeutic capabilities demonstrate the usefulness of flavonoids in producing drugs related to cancer and the nervous system.

Various physicochemical and structural properties of flavonoid can be attributed to differences in activity and can be found in physicochemical characteristics, including H bond donors, H bond acceptors, topological polarity surface area and water-lipid partition coefficients, because the proper solubility and water lipid partition coefficient play an important role in the effectiveness of the drug.

Since flavonoids contain the same skeleton, the functional differences are mainly related to the replacement groups. The relationship between the chemical constitution fragments and the biological effects suggests that significantly different side chains can influence flavonoid activity in the same target. Apart from general biological functions, the specific functions of the various subclasses of flavonoids were analyzed and demonstrated at the target and pathway levels. For example, flavones and isoflavones were significantly amplified in a pathway associated with more cancers than others, suggesting potential therapeutic benefits in treating cancer. Flavan-3-oles have also been found in cellular processing and lymphocyte regulation, flavones have a specific effect on cardiovascular activity, and isoflavones are closely related to cellular multisystem disorders.

Cumulative structure activity relationship findings from previous pharmacological reports provide useful evidence for the role of different functional groups in nutritional benefits. Based on the description above, it can be concluded that the 4-carbonyl group, the C_2_ =C_3_ double bond, and the hydroxylation pattern, especially the 3-OH and catechol residue in the B ring, are the main known factors of the therapeutical effects of flavonoids. For example, the beneficial effect of hydroxylation is achieved in terms of exclusive antiviral, antibacterial, cardioprotective, anti-diabetic and carcinogenic effects. O-methylation is useful for antiviral, antibacterial, anti-diabetic, but of lower benefit for anti-inflammatory and anti-cancer effects. In general, glycosylation can reduce the associated activity as anti-Alzheimer's disease, but on the contrary increases the antiviral and antibacterial effects.

However, future approaches and further research efforts at the clinical level and in the field of bioavailability will provide a deeper understanding of the therapeutic effects of flavonoids on human health in general.

## Conflict of interests

The author declares that they do not have any conflict of interests.

## Declaration of Competing Interest

The authors declare that they have no known competing financial interests or personal relationships that could have appeared to influence the work reported in this paper.

## References

[1] Abbas H., Abou Baker D. (2020). Biological evaluation of selenium nanoparticles biosynthesized by Fusarium semitectum as antimicrobial and anticancer agents. Egypt. J. Chem..

[2] Abdelwahab S.I., Mohan S., Abdulla M.A., Sukari M.A., Abdul A.B., Taha M.M.E., Syam S., Ahmad S., Lee K.H. (2011). The methanolic extract of Boesenbergia rotunda (L.) Mansf. and its major compound pinostrobin induces anti-ulcerogenic property in vivo: possible involvement of indirect antioxidant action. J. Ethnopharmacol..

[3] Abou Baker D.H., Al-Moghazy M., ElSayed A.A.A. (2020). The in vitro cytotoxicity, antioxidant and antibacterial potential of *Satureja hortensis* L. essential oil cultivated in Egypt. Bioorg. Chem..

[4] Abou Baker D.H., Rady H.M. (2020). Bioassay-guided approach employed to isolate and identify anticancer compounds from *Physalis peruviana* calyces. Plant Arch..

[5] Abou Baker D.H. (2020). Plants against Helicobacter pylori to combat resistance: an ethnopharmacological review. Biotechnol. Rep..

[6] Abou Baker D.H. (2020). *Achillea millefolium* L. ethyl acetate fraction induces apoptosis and cell cycle arrest in human cervical cancer (HeLa) cells. Ann. Agric. Sci.

[7] Abou Baker D.H., Ibrahim B.M., Hassan N.S., Yousuf A.F., El Gengaihi S. (2020). Exploiting *Citrus aurantium* seeds and their secondary metabolites in the management of Alzheimer disease. Toxicol. Rep..

[9] Akbari A., Majd H.M., Rahnama R., Heshmati J., Morvaridzadeh M., Agah S., Amini S.M., Masoodi M. (2020). Cross-talk between oxidative stress signaling and microRNA regulatory systems in carcinogenesis: focused on gastrointestinal cancers. Biomed. Pharmacother..

[10] Allam S.F., Soudy B.A.N., Hassan A.S., Ramadan M.M., Abou Baker D.H. (2018). How do mentha plants induce resistance against *Tetranychus urticae* (Acari: Tetranychidae) in organic farming?. J. Plant Prot. Res..

[11] Aly A.M., Al-Alousi L., Salem H.A. (2005). Licorice: a possible anti-inflammatory and anti-ulcer drug. Aaps Pharmscitech..

[12] Ammar M.I., Nenaah G.E., Mohamed A.H.H. (2013). Antifungal activity of prenylated flavonoids isolated from Tephrosia apollinea L. against four phytopathogenic fungi. Crop Prot..

[13] Amrutha K., Nanjan P., Shaji S.K. (2014). Discovery of lesser known flavones as inhibitors of NF-kappaB signaling in MDA-MB-231 breast cancer cells – a SAR study. Bioorg. Med. Chem. Lett..

[15] Ares J.J., Outt P.E., Randall J.L., Johnston J.N., Murray P.D., O’Brien L.M., Weisshaar P.S., Ems B.L. (1996). Synthesis and biological evaluation of flavonoids and related compounds as gastroprotective agents. Bioorg. Med. Chem. Lett..

[16] Ares J.J., Outt P.E., Randall J.L., Murray P.D., Weisshaar P.S., O’Brien L.M., Ems B.L., Kakodkar S.V., Kelm G.R. (1995). Synthesis and biological evaluation of substituted flavones as gastroprotective agents. J. Med. Chem..

[17] Asha M.K., Debraj D., Edwin J.R., Srikanth H.S., Muruganantham N., Dethe S.M., Anirban B., Jaya B., Deepak M., Agarwal A. (2013). In vitro anti-Helicobacter pylori activity of a flavonoid rich extract of Glycyrrhiza glabra and its probable mechanisms of action. J. Ethnopharmacol..

[18] Asmat U., Abad K., Ismail K. (2016). Diabetes mellitus and oxidative stress—A concise review. Saudi Pharmac. J..

[19] Áy É., Hunyadi A., Mezei M., Minárovits J., Hohmann J. (2019). Flavonol 7-O-Glucoside Herbacitrin inhibits HIV-1 replication through simultaneous integrase and reverse transcriptase inhibition. Evid.-based Complement. Altern. Med..

[20] Babajide O.J., Babajide O.O., Daramola A.O., Mabusela W.T. (2008). Flavonols and an oxychromonol from Piliostigma reticulatum. Phytochemistry.

[21] Babu P.V.A., Liu D., Gilbert E.R. (2013). Recent advances in understanding the anti-diabetic actions of dietary flavonoids. J. Nutr. Biochem..

[22] Bae M.J., Shin H.S., See H.J., Jung S.Y., Kwon D.A., Shon D.H. (2016). Baicalein induces CD4+ Foxp3+ T cells and enhances intestinal barrier function in a mouse model of food allergy. Sci. Rep..

[23] Baghel S.S., Shrivastava N., Baghel R.S., Agrawal P., Rajput S. (2012). A review of quercetin: antioxidant and anticancer properties. World J. Pharm. Pharm. Sci..

[24] Bagli E., Stefaniotou M., Morbidelli L., Ziche M., Psillas K., Murphy C., Fotsis T. (2004). Luteolin inhibits vascular endothelial growth factor-induced angiogenesis; inhibition of endothelial cell survival and proliferation by targeting phosphatidylinositol 3′-kinase activity. Cancer Res..

[25] Bahramsoltani R.F.M., Farahani M.S., Rahimi R. (2015). Phytochemical constituents as future antidepressants: a comprehensive review. Rev. Neurosci..

[27] Bartekova M., Ferenczyova K., Radosinska J., Pancza D., Barancik M., Ravingerova T. (2018). Cardioprotective effects of acute and chronic treatment with flavonoid quercetin against ischemia/reperfusion injury in isolated rat hearts: focus on the role of age in the efficiency of treatment. J. Mol. Cell. Cardiol..

[28] Bauer D., Redmon N., Mazzio E., Soliman K.F. (2017). Apigenin inhibits TNFa/IL-1a-induced CCL2 release through IKBK-epsilon signaling in MDA-MB-231 human breast cancer cells. PLoS One.

[29] Belofsky G., Kolaczkowski M., Adams E., Schreiber J., Eisenberg V., Coleman C.M., Zou Y., Ferreira D. (2013). Fungal ABC transporter-associated activity of isoflavonoids from the root extract of daleaformosa. J. Nat. Prod..

[30] Beserra F.P., Rozza A.L., Vieira A.J., Gushiken L.F.S., Pellizzon C.H. (2016).

[31] Bian C., Xu T., Zhu H., Pan D., Liu Y., Luo Y., Wu P., Li D. (2015). Luteolin inhibits Ischemia/reperfusion-induced myocardial injury in rats via downregulation of microRNA-208b-3p. PLoS One.

[33] Brakenhielm E., Cao R., Cao Y. (2001). Suppression of angiogenesis, tumor growth, and wound healing by resveratrol, a natural compound in red wine and grapes. FASEB J..

[36] Butterweck V. (2003). Mechanism of action of St John’s wort in depression. CNS Drugs.

[37] Cantelli B.A.M., Bitencourt T.A., Komoto T.T., Beleboni R.O., Marins M., Fachin A.L. (2017). Caffffeic acid and licochalcone A interfere with the glyoxylate cycle of Trichophyton rubrum. Biomed. Pharmacother..

[38] Cao Y., Dai B., Wang Y., Huang S., Xu Y., Cao Y., Gao P., Zhu Z., Jiang Y. (2008). In vitro activity of baicalein against Candida albicans biofilms. Int. J. Antimicrob. Agents.

[40] Cassetta A., Stojan J., Krastanova I., Kristan K., BrunskoleŠvegelj M., Lamba D., LanišnikRižner T. (2017). Structural basis for inhibition of 17_-hydroxysteroid dehydrogenases by phytoestrogens: The case of fungal17_-HSDcl. J. Steroid Biochem. Mol. Biol..

[41] Cassidy L., Fernandez F., Johnson J.B., Naiker M., Owoola A.G., Broszczak D.A. (2020). Oxidative stress in alzheimer’s disease: A review on emergent natural polyphenolic therapeutics. Complement. Ther. Med..

[42] Castillo-Juárez I., González V., Jaime-Aguilar H., Martínez G., Linares E., Bye R., Romero I. (2009). Anti-Helicobacter pylori activity of plants used in Mexican traditional medicine for gastrointestinal disorders. J. Ethnopharmacol..

[43] Chattopadhyay D., Naik T.N. (2007). Antivirals of ethnomedicinal origin: structure-activity relationship and scope. Mini Rev. Med. Chem..

[44] Cheema H.S., Prakash O., Pal A., Khan F., Bawankule D.U., Darokar M.P. (2014). Glabridin induces oxidative stress mediated apoptosis like cell death of malaria parasite Plasmodium falciparum. Parasitol. Int..

[45] Chen Y.C., Yang L.L., Lee T.J.F. (2004). Oroxylin A inhibition of lipopolysaccharide-induced iNOS and COX-2 gene expression via suppression of nuclear factor-kB activation. BiochemPharmacol.

[46] Chen C., Zhou J., Ji C. (2010). Quercetin: a potential drug to reverse multidrug resistance. Life Sci..

[47] Chen C.N., Lin C.P., Huang K.K., Chen W.C., Hsieh H.P., Liang P.H., Hsu J.T.A. (2005). Inhibition of SARS-CoV 3C-like protease activity by theaflavin-3, 3’-digallate (TF3). Evid.-based Complement. Altern. Med..

[48] Chen L., Li J., Luo C., Liu H., Xu W., Chen G., Liew O.W., Zhu W., Puah C.M., Shen X., Jiang H. (2006). Binding interaction of quercetin-3-β-galactoside and its synthetic derivatives with SARS-CoV 3CLpro: Structure–activity relationship studies reveal salient pharmacophore features. Bioorg. Med. Chem..

[49] Cho J.K., Curtis-Long M.J., Lee K.H., Kim D.W., Ryu H.W., Yuk H.J., Park K.H. (2013). Geranylated flavonoids displaying SARS-CoV papain-like protease inhibition from the fruits of Paulownia tomentosa. Bioorg. Med. Chem..

[50] Chojnacka K., Witek-Krowiak A., Skrzypczak D., Mikula K., Młynarz P. (2020). Phytochemicals containing biologically active polyphenols as an effective agent against Covid-19-inducing coronavirus. J. Funct. Foods.

[51] Coelho R.G., Batista L.M., Santos L.C.D., Brito A.R.M.D.S., Vilegas W. (2006). Phytochemical study and antiulcerogenic activity of Syngonanthus bisulcatus (Eriocaulaceae). Rev Bras. C. Farm..

[52] Cos P., Ying L., Calomme M., Hu J.P., Cimanga K., Van Poel B. (1998). Structure–activity relationship and classification of flavonoids as inhibitors of xantine oxidase and superoxide scavengers. J. Nat. Prod..

[53] Costa G., Francisco V., Lopes M.C., Cruz M.T., Batista M.T. (2012). Intracellular signaling pathways modulated by phenolic compounds: Application for new anti-inflammatory drugs discovery. Curr. Med. Chem..

[54] Cushnie T.T., Lamb A.J. (2011). Recent advances in understanding the antibacterial properties of flavonoids. Int. J. Antimicrob. Agents.

[57] da Silva C.R., de Andrade Neto J.B., de Sousa Campos R., Figueiredo N.S., Sampaio L.S., Magalhães H.I.F., Cavalcanti B.C., Gaspar D.M., de Andrade G.M., Lima I.S.P. (2013). Synergistic effect of the flavonoid catechin, quercetin, or epigallocatechin gallate with fluconazole induces apoptosis in Candida tropicalis resistant to fluconazole. Antimicrob. Agents Chemother..

[58] Da X., Nishiyama Y., Tie D., Hein K.Z., Yamamoto O., Morita E. (2019). Antifungal activity and mechanism ofaction of Ou-gon (Scutellaria root extract) components against pathogenic fungi. Sci. Rep..

[60] de la Peña J.B.1K.C., Lee H.L., Yoon S.Y., Kim H.J., Hong E.Y., Kim G.H., Ryu J.H., Lee Y.S., Kim K.M., Cheong J.H. (2014). Luteolin mediates the antidepressant-like effects of Cirsium japonicum in mice, possibly through modulation of the GABAA receptor. Arch Pharm. Res..

[61] Del Bo’ C., Roursgaard M., Porrini M., Loft S., Møller P., Riso P. (2016). Different effects of anthocyanins and phenolic acids from wild blueberry (Vaccinium angustifolium) on monocytes adhesion to endothelial cells in a TNF‐α stimulated proinflammatory environment. Mol. Nutr. Food Res..

[62] Devi K.P., Malar D.S., Nabavi S.F., Sureda A., Xiao J., Nabavi S.M., Daglia M. (2015). Kaempferol and inflammation: from chemistry to medicine. Pharmacol. Res..

[63] Duan F., Xin G., Niu H., Huang W. (2017). Chlorinated emodin as a natural antibacterial agent against drug-resistant bacteria through dual influence on bacterial cell membranes and DNA. Sci. Rep..

[64] Duda-Madej A., Kozłowska J., Krzyżek P., Anioł M., Seniuk A., Jermakow K., Dworniczek E. (2020). Antimicrobial o-alkyl derivatives of naringenin and their oximes against multidrug-resistant bacteria. Molecules.

[65] Echeverría J., Opazo J., Mendoza L., Urzúa A., Wilkens M. (2017). Structure‐activity and lipophilicity relationships of selected antibacterial natural flavones and flavanones of Chilean flora. Molecules.

[66] El Gengaihi S.E., Arafa M.M., Abou Baker D.H., Shoaib R.M., Asker M.S., Abdelhamid S.A., Hassan E.M. (2020). Chemical, biological and molecular studies on different citrus species wastes. Plant Arch..

[67] El-Gengaihi S.E., Mossa A.T.H., Refaie A.A., Abou Baker D.H. (2016). Hepatoprotective efficacy of *Cichorium intybus* L. extract against carbon tetrachloride-induced liver damage in rats. J. Diet Suppl..

[68] El-Gengaihi S.E., Hamed M.A., Abou Baker D.H., Mossa A.T.H. (2016). Flavonoids from sugar beet leaves as hepatoprotective agent. Int. J. Pharm. Pharm. Sci..

[70] Ferrali M., Signorini C., Caciotti B., Sugherini L., Ciccoli L., Giachetti D. (1997). Protection against oxidative damage of erythrocyte membranes by the flavonoid quercetin and its relation to iron chelating activity. FEBS Lett..

[71] Fukai T., Marumo A., Kaitou K., Kanda T., Terada S., Nomura T. (2002). Anti-Helicobacter pylori flavonoids from licorice extract. Life Sci..

[73] Gibellini L., Bianchini E., De Biasi S., Nasi M., Cossarizza A., Pinti M. (2015). Natural compounds modulatingmitochondrial functions. Evid. Based Complement. Altern. Med..

[75] Gledhill J.R., Walker J.E. (2005). Inhibition sites in F1-ATPase from bovine heart mitochondria. Biochem. J..

[76] Górniak I., Bartoszewski R., Króliczewski J. (2019). Comprehensive review of antimicrobial activities of plant flavonoids. Phytochem. Rev..

[77] Guan L.-P., Liu B.-Y. (2016). Antidepressant-like effects and mechanisms of flavonoids and related analogues. Eur. J. Med. Chem..

[78] Guntuku L., Naidu V.G.M., Ganesh Yerra V. (2016). Mitochondrial dysfunction in gliomas: pharmacotherapeuticpotential of natural compounds. Curr. Neuropharmacol..

[79] Guo Y., Liu Y., Zhang Z., Chen M., Zhang D., Tian C., Liu M., Jiang G. (2020). The antibacterial activity and mechanism of action of luteolin against Trueperella pyogenes. Infect. Drug Resist..

[80] Guti´errez-del-Río I., Villar C.J., Lomb´o F., Garde- Cerd´an T., Gonzalo-Diago A. (2016). Kaempferol: Biosynthesis, Food Sources and Therapeutic Uses.

[81] Ha J., Zhao L., Zhao Q., Yao J., Zhu B.B., Lu N. (2012). Oroxylin A improves the sensitivity of HT-29 human colon cancer cells to 5- FU through modulation of the COX-2 signaling pathway. Biochem. Cell. Biol..

[82] Habauzit V., Morand C. (2012). Evidence for a protective effect of polyphenols-containing foods on cardiovascular health: an update for clinicians. Ther. Adv Chronic Dis..

[83] Hahm K.B., Song Y.J., Oh T.Y., Lee J.S., Surh Y.J., Kim Y.B., Yoo B.M., Kim J.H., Ha S.U., Nahm K.T., Kim M.W. (2003). Chemoprevention of Helicobacter pylori-associated gastric carcinogenesis in a mouse model; Is it possible?. BMB Rep..

[85] Han X.H., H S., Hwang J.S. (2007). Monoamine oxidase inhibitory components from Cayratia japonica. Arch. Pharm. Res..

[86] Haraguchi H., Tanimoto K., Tamura Y., Mizutani K., Kinoshita T. (1998). Mode of antibacterial action of retrochalcones from Glycyrrhiza inflata. Phytochemistry.

[87] Hayes J.D., Dinkova-Kostova A.T., Tew K.D. (2020). Oxidative stress in cancer. Cancer Cell.

[88] He Y., Xia Z., Yu D., Wang J., Jin L., Huang D., Ye X., Li X., Zhang B. (2019). Hepatoprotective effects and structure-activity relationship of five flavonoids against lipopolysaccharide/d-galactosamine induced acute liver failure in mice. Int. Immunopharmacol..

[89] Heim K.E., Tagliaferro A.R., Bobilya D.J. (2002). Flavonoid antioxidants: chemistry, metabolism and structure–activity relationships. J. Nutr. Biochem..

[90] Heimfarth L., da Silva Nascimento L., da Silva M.D.J.A., de Lucca Junior W., Lima E.S., Quintans-Junior L.J., da Veiga-Junior V.F. (2021). Neuroprotective and anti-inflammatory effect of pectolinarigenin, a flavonoid from Amazonian Aegiphila integrifolia (Jacq.), against lipopolysaccharide-induced inflammation in astrocytes via NFκB and MAPK pathways. Food Chem Toxicol..

[91] Hernández-Aquino E., Muriel P. (2018). Beneficial effects of naringenin in liver diseases: molecular mechanisms. World J. Gastroenterol..

[92] Hirano R., Sasamoto W., Matsumoto A., Itakura H., Igarashi O., Kondo K. (2001). Antioxidant ability of various flavonoids against DPPH radicals and LDL oxidation. J. Nutr. Sci. Vitaminol..

[93] Hou W.C.L., R D., Chen C.T., Lee M.H. (2005). Monoamine oxidase B (MAO-B) inhibition by active principles from Uncaria rhynchophylla. J. Ethnopharmacol..

[94] Hou X.M., Zhang M.S., Qin X.J. (2017). Vasodilation of quercetin on rat renal artery and the relationship with L-type voltage-gated Ca2+ channels and protein kinase C. Sheng li xue bao: [Acta Physiol. Sin.].

[96] Huang Z., Fang F., Wang J. (2010). Structural activity relationship of flavonoids with estrogen-related receptor gamma. FEBS Lett..

[97] Huang S., Cao Y.Y., Dai B.D., Sun X.R., Zhu Z.Y., Cao Y.B., Wang Y., Gao P.H., Jiang Y.Y. (2008). In vitrosynergism of fluconazole and baicalein against clinical isolates of Candida albicans resistant to fluconazole. Biol. Pharm. Bull..

[98] Hugel H.M., Jackson N., May B., Zhang A.L., Xue C.C. (2016). Polyphenol protection and treatment of hypertension. Phytomedicine.

[99] Hwang J.S.L., S A., Hong S.S., Lee K.S., Lee M.K., Hwang B.Y., Ro J.S. (2005). Monoamine oxidase inhibitory components from the roots of Sophora flflavescens. Arch. Pharmacal. Res..

[100] Ibrahim B.M.M., Salamaa Abeer A.A., Yassina Nemat A., Mahmouda Sawsan S., El-Dinb Amina A.Gamal, Shaffieb Nermeen A., Abou Baker Doha H. (2020). Potential effects of glimepiride and a herbal mixture on hyperglycaemia, hypercholesterolaemia and oxidative stress. Plant Arch..

[101] Ibrahim E.A., Abou Baker D.H., El-Baz F.K. (2016). Anti-inflammatory and antioxidant activities of rhubarb roots extract. Int. J. Pharm. Sci. Rev. Res..

[102] Ibrahim E.A., Baker D.H., El-Baz F.K. (2016). Anti-inflammatory and antioxidant activities of rhubarb roots extract. Int. J. Pharm. Sci. Rev. Res..

[103] Ibrahim N.H., Awaad A.S., Alnafisah R.A., Alqasoumi S.I., El-Meligy R.M., Mahmoud A.Z. (2018). In–Vitro activity of Desmostachya bipinnata (L.) Stapf successive extracts against Helicobacter pylori clinical isolates. Saudi Pharm. J..

[104] Ilboudo O., Bonzi S., Tapsoba I., Somda I., Bonzi-Coulibaly Y.L. (2016). In vitro antifungal activity of flavonoid diglycosides of Mentha piperita and their oxime derivatives against two cereals fungi. C. R. Chim..

[105] Im H.I., Joo W.S., Nam E., Lee E.S., Hwang Y.J., Kim Y.S. (2005). Baicalein prevents 6-hydroxydopamine-induced dopaminergic dysfunction and lipid peroxidation in mice. J. Pharmacol. Sci.

[106] Imran M., Rauf A., Abu-Izneid T., Nadeem M., Shariati M.A., Khan I.A., Imran A., Orhan I.E., Rizwan M., Atif M., Gondal T.A. (2019). Luteolin, a flavonoid, as an anticancer agent: a review. Biomed. Pharmacother..

[107] Ishisaka M.1, K K., Yamauchi M., Tsuruma K., Shimazawa M., Tsuruta A., Hara H. (2011). Luteolin shows an antidepressant-like effect via suppressing endoplasmic reticulum stress. Biol. Pharm. Bull..

[109] Lee J.S., Kim H.S., Hahm K.B., Sohn M.W., Yoo M., Johnson J.A., Surh Y.J. (2007). Ann. N. Y. Acad. Sci..

[110] Jadhav S.G., Meshram R.J., Gond D.S., Gacche R.N. (2013). Inhibition of growth of *Helicobacter pylori* and its urease by coumarin derivatives: molecular docking analysis. J Pharmacy Res..

[111] Jäger W., Zembsch B., Wolschann P., Pittenauer E., Sausville E.A., Sedlacek H.H., Graf J., Thalhammer T. (1998). Metabolism of the anticancer drug flavopiridol, a new inhibitor of cyclin dependent kinases, in rat liver. Life Sci..

[112] Jaguezeski A.M., Perin G., Crecencio R.B., Baldissera M.D., Stefanil L.M., da Silva A.S. (2018). Addition of curcumin in dairy sheep diet in the control of subclinical mastitis. Acta Sci. Vet..

[113] Jain D.L., Baheti A.M., Parakh S.R., Ingale S.P., Ingale P.L. (2007). PHCOG MAG.: research Article Study of antacid and diuretic activity of ash and extracts of Musa sapientum L. fruit peel. Phcog. Mag..

[115] Ji L., Du Q., Li Y., Hu W. (2016). Puerarin inhibits the inflammatory response in atherosclerosis via modulation of the NF-κB pathway in a rabbit model. Pharmacol. Rep..

[117] Jiang H., Xia Q., Wang X., Song J., Bruce I.C. (2005). Luteolin induces vasorelaxion in rat thoracic aorta via calcium and potassium channels. Pharmazie.

[119] Jo S., Kim S., Shin D.H., Kim M.S. (2020). Inhibition of SARS-CoV 3CL protease by flavonoids. J. Enzyme Inhib. Med. Chem..

[120] Kang K., Fong W.-P., Tsang P.W.-K. (2010). Antifungal activity of baicaleinagainst Candida krusei does not involve apoptosis. Mycopathologia.

[121] Kasala E.R., Bodduluru L.N., Madana R., Gogoi R., Barua C.C. (2015). Chemopreventive and therapeutic potential of chrysin in cancer: mechanistic perspectives. Toxicol. Lett..

[122] Katalinić M., Rusak G., Barović J.D., Šinko G., Jelić D., Antolović R., Kovarik Z. (2010). Structural aspects of flavonoids as inhibitors of human butyrylcholinesterase. Eur. J. Med. Chem..

[123] Khaerunnisa, S., Kurniawan, H., Awaluddin, R., Suhartati, S. and Soetjipto, S., 2020. Potential inhibitor of COVID-19 main protease (Mpro) from several medicinal plant compounds by molecular docking study. Prepr. doi10, 20944, pp.1–14.

[124] Khan H., Amin S., Kamal M.A., Patel S. (2018). Flavonoids as acetylcholinesterase inhibitors: current therapeutic standing and future prospects. Biomed. Pharmacother..

[125] Kim J.E., Kwon J.Y., Seo S.K., Son J.E., Jung S.K., Min S.Y. (2010). Cyanidin suppresses ultraviolet B-induced COX-2 expression in epidermal cells by targeting MKK4, MEK1, and Raf-1. Biochem. Pharmacol..

[126] Kim H.P., Son K.H., Chang H.W., Kang S.S. (2004). Anti-inflammatory plant flavonoids and cellular action mechanisms. J. Pharmacol. Sci..

[127] Kim M., Lim S.J., Kang S.W., Um B.H., Nho C.W. (2014). Aceriphyllum rossii extract and its active compounds, quercetin and kaempferol inhibit IgE-mediated mast cell activation and passive cutaneous anaphylaxis. J. Agric. Food Chem..

[128] Kim P.K., Son K.H., Chang H.W., Kang S.S. (2004). Anti-inflammatory plant flavonoids and cellular action mechanisms. J. Pharmacol. Sci..

[129] Kim S., Woo E.R., Lee D.G. (2019). Synergistic antifungal activity of isoquercitrin: apoptosis and membrane permeabilization related to reactive oxygen species in Candida albicans. IUBMB life.

[130] Kimata M., Shichijo M., Miura T., Serizawa I., Inagaki N., Nagai H. (2000). Effects of luteolin, quercetin and baicalein on immunoglobulin E-mediated mediator release from human cultured mast cells. Clin. Exp. Allergy.

[131] Kluknavsky M., Balis P., Puzserova A., Radosinska J., Berenyiova A., Drobna M., Lukac S., Muchova J., Bernatova I. (2016). (−)-Epicatechin prevents blood pressure increase and reduces locomotor hyperactivity in young spontaneously hypertensive rats. Oxid. Med. Cell. Longev..

[132] Krych J., Gebicka L. (2013). Catalase is inhibited by flavonoids. Int. J. Biol. Macromol..

[133] Krych J., Gebicka L. (2013). Catalase is inhibited by flflavonoids. Int. J. Biol. Macromol..

[135] Kvasnickova E., Matatkova O., Cejkova A., Masak J. (2015). Evaluation of baicalein, chitosan and usnic acid effect on Candida parapsilosis and Candida krusei biofilm using a Cellavista device. J. Microbiol. Methods.

[136] La Casa C., Villegas I., De La Lastra C.A., Motilva V., Calero M.M. (2000). Evidence for protective and antioxidant properties of rutin, a natural flavone, against ethanol induced gastric lesions. J. Ethnopharmacol..

[137] Lago J.H.G., Toledo-Arruda A.C., Mernak M., Barrosa K.H., Martins M.A., Tibério I.F., Prado C.M. (2014). Structure-activity association of flavonoids in lung diseases. Molecules.

[138] Lättig J., Böhl M., Fischer P., Tischer S., Tietböhl C., Menschikowski M., Gutzeit H.O., Metz P., Pisabarro M.T. (2007). Mechanism of inhibition of human secretory phospholipase A2 by flavonoids: rationale for lead design. J. Comput.-aided Mol. Des..

[139] Lee H.S., Cho H.J., Yu R., Lee K.W., Chun H.S., Park J.H.Y. (2014). Mechanisms underlying apoptosis-inducing effects of kaempferol in HT- 29 human colon cancer cells. Int. J. Mol. Sci..

[140] Lee J.I., Hsu B.H., Wu D., Barrett J.S. (2006). Separation and characterization of silybin, isosilybin, silydianin and silychristin in milk thistle extract by liquid chromatography electrospray tandem mass spectrometry. J. Chromatogr. A.

[142] Lee H., Woo E.R., Lee D.G. (2018). Apigenin induces cell shrinkage in Candida albicans by membrane perturbation. FEMS Yeast Res..

[144] Lee M.H.L., R D., Shen L.Y., Yang L.L., Yen K.Y., Hou W.C. (2001). Monoamine oxidase B and free radical scavenging activities of natural flflavonoids in Melastoma candidum D. Don. J. Agric. Food. Chem.

[145] Lewis D.A., Shaw G.P. (2001). A natural flavonoid and synthetic analogues protect the gastric mucosa from aspirin-induced erosions. J. Nutr. Biochem..

[146] Li Y.Q., Li Z.L., Zhao W.J., Wen R.X., Meng Q.W., Zeng Y. (2006). Synthesis of stilbene derivatives with inhibition of SARS coronavirus replication. Eur. J. Med. Chem..

[147] Li B.Q., Fu T., Dongyan Y., Mikovits J.A., Ruscetti F.W., Wang J.M. (2000). Flavonoid baicalin inhibits HIV-1 infection at the level of viral entry. Biochem. Biophys. Res. Commun..

[148] Li Y., Chang W., Zhang M., Li X., Jiao Y., Lou H. (2015). Synergistic and drug-resistant reversing effects ofdiorcinol D combined with fluconazole against Candida albicans. FEMS Yeast Res..

[150] Y.F. Li, M. Y, L. Yuan, Antidepressant Effffects of Quercertin-3-apiosyl (1→2)- [rhamnosyl (1→6)]-glucoside in Mice, vol 14, (2000). pp. 1125–1127.

[153] Liu B.1, X C., Wu X.1, Liu F.1, Du Y.1, Sun J.1, Tao J.3, Dong J.4 (2015). icariin exerts an antidepressant effect in an unpredictable chronic mild stress model of depression in rats and is associated with the regulation of hippocampal neuroinflammation. Neuroscience.

[154] Liu C., Zhu L., Fukuda K., Ouyang S., Chen X., Wang C., Zhang C.J., Martin B., Gu C., Qin L., Rachakonda S. (2017). The flavonoid cyanidin blocks binding of the cytokine interleukin-17A to the IL-17RA subunit to alleviate inflammation in vivo. Sci. Signal..

[155] Liu W., Li L.P., Zhang J.D., Li Q., Shen H., Chen S.M., He L.J., Yan L., Xu G.T., An M.M. (2014). Synergistic antifungal effect of glabridin and fluconazole. PLoS One.

[156] Liu Y., Niu L., Cui L., Hou X., Li J., Zhang X., Zhang M. (2014). Hesperetin inhibits rat coronary constriction by inhibiting Ca2+ influx and enhancing voltage-gated K+ channel currents of the myocytes. Eur. J. Pharmacol..

[157] Lotito S.B., Frei B. (2006). Consumption of flavonoid-rich foods and increase plasma antioxidant capacity in humans: cause, consequence, or epiphenomenon?. Free Radic. Biol. Med..

[158] Lu J., Papp L.V., Fang J., Rodriguez-Nieto S., Zhivotovsky B., Holmgren A. (2006). Inhibition of mammalian thioredoxin reductase by some flavonoids: implications for myricetin and quercetin anticancer activity. Cancer Res..

[159] Lung J., Lin Y.S., Yang Y.H., Chou Y.L., Shu L.H., Cheng Y.C., Liu H.T., Wu C.Y. (2020). The potential chemical structure of anti‐SARS‐CoV‐2 RNA‐dependent RNA polymerase. J. Med. Virol..

[160] Luo Y., Wang S.X., Zhou Z.Q., Wang Z., Zhang Y.G., Zhang Y. (2014). Apoptotic effect of genistein on human colon cancer cells via inhibiting the nuclear factor-kappa B (NF- kB) pathway. Tumour Biol.

[161] Lv QQ1 W.W., Guo X.L., Liu R.L., Yang Y.P., Zhou D.S., Zhang J.X., Liu J.Y. (2014). Antidepressant activity of astilbin: involvement of monoaminergic neurotransmitters and BDNF signal pathway. Biol. Pharm. Bull..

[162] Maaliki D., Shaito A.A., Pintus G., El-Yazbi A., Eid A.H. (2019). Flavonoids in hypertension: a brief review of the underlying mechanisms. Curr. Opin. Pharmacol..

[163] Macready A.L., George T.W., Chong M.F., Alimbetov D.S., Jin Y., Vidal A. (2014). Flavonoid-rich fruit and vegetables improve microvascular reactivity and inflammatory status in men at risk of cardiovascular disease—flavurs: a randomized controlled trial. Am. J. Clin. Nutr..

[164] Mafioleti L., da Silva Junior I.F., Colodel E.M., Flach A., de Oliveira Martins D.T. (2013). Evaluation of the toxicity and antimicrobial activity of hydroethanolic extract of Arrabidaea chica (Humb. & Bonpl.) B. Verl. J. Ethnopharmacol..

[165] Magalingam K.B., Radhakrishnan A.K., Haleagrahara N. (2015). Protective mechanisms of flavonoids in Parkinson’s disease. Oxid. Med. Cell. Longev..

[166] Majewska-Wierzbicka M., Czeczot H. (2012). Flavonoids in the prevention and treatment of cardiovascular diseases. Pol Merkur Lekarski.

[167] Manoharan R.K., Lee J.-H., Kim Y.-G., Lee J. (2017). Alizarin and chrysazin inhibit biofilm and hyphal formationby Candida albicans. Front. Cell. Infect. Microbiol..

[168] Marin L., Miguelez E.M., Villar C.J., Lomb´o F. (2015). Bioavailability of dietary polyphenols and gut microbiota metabolism: antimicrobial properties. Biomed. Res. Int..

[169] Martini S., D’Addario C., Colacevich A., Focardi S., Borghini F., Santucci A., Figura N., Rossi C. (2009). Antimicrobial activity against Helicobacter pylori strains and antioxidant properties of blackberry leaves (Rubus ulmifolius) and isolated compounds. Int. J. Antimicrob. Agents.

[170] Masilamani M., Wei J., Bhatt S., Paul M., Yakir S., Sampson H.A. (2011). Soybean isoflavones regulate dendritic cell function and suppress allergic sensitization to peanut. J. Allergy Clin. Immunol..

[171] Matsubara S., Shibata H., Ishikawa F., Yokokura T., Takahashi M., Sugimura T., Wakabayashi K. (2003). Suppression of Helicobacter pylori-induced gastritis by green tea extract in Mongolian gerbils. Biochem. Biophys. Res. Commun..

[172] Mbaveng A.T., Ngameni B., Kuete V., Simo I.K., Ambassa P., Roy R., Abegaz B.M. (2008). Antimicrobial activity of the crude extracts and five flavonoids from the twigs of Dorstenia barteri (Moraceae). J. Ethnopharmacol..

[173] Mbaveng A.T., Ngameni B., Kuete V., Simo I.K., Ambassa P., Roy R., Abegaz B.M. (2008). Antimicrobial activity of the crude extracts and five flavonoids from the twigs of *Dorstenia barteri* (Moraceae). J. Ethnopharmacol..

[174] Mbaveng A.T., Kuete V., Ngameni B., Beng V.P., Ngadjui B.T., Meyer J.J.M., Lall N. (2012). Antimicrobialactivities of the methanol extract and compounds from the twigs of Dorsteniamannii (Moraceae). BMC Compl. Altern. Med.

[175] Meneguzzo F., Ciriminna R., Zabini F., Pagliaro M. (2020). Review of evidence available on hesperidin-rich products as potential tools against COVID-19 and hydrodynamic cavitation-based extraction as a method of increasing their production. Processes.

[176] Ming D., Wang D., Cao F., Xiang H., Mu D., Cao J., Li B., Zhong L., Dong X., Zhong X., Wang L. (2017). Kaempferol inhibits the primary attachment phase of biofilm formation in Staphylococcus aureus. Front. Microbiol..

[179] Moazeni M., Hedayati M.T., Nabili M., Mousavi S.J., AbdollahiGohar A., Gholami S. (2017). Glabridin triggersover-expression of MCA1 and NUC1 genes in Candida glabrata: Is it an apoptosis inducer?. J. Mycol. Méd..

[180] Mohamed T., Rao P.P. (2010). Corrigendum to ‘‘Design, synthesis and evaluation of 2, 4-disubstituted pyrimidines as cholinesterase inhibitors”[Bioorg. Med. Chem. Lett. 20. Bioorg. Med. Chem. Lett..

[181] Mohan S., Nandhakumar L. (2014). Role of various flavonoids: hypotheses on novel approach to treat diabetes. J. Med. Hypoth. Ideas.

[183] Morris J., Moseley V.R., Cabang A.B., Coleman K., Wei W., Garrett- Mayer E. (2016). Reduction in promotor methylation utilizing EGCG (Epigallocatechin-3-gallate) restores RXRalpha expression in human colon cancer cells. Oncotarget.

[184] Mossa A.T.H., Ibrahim F.M., Mohafrash S.M., Abou Baker D.H., El Gengaihi S. (2015). Protective effect of ethanolic extract of grape pomace against the adverse effects of cypermethrin on weanling female rats. Evid. Based Complement. Altern. Med..

[185] Moudgal V., Sobel J. (2010). Antifungals to treat Candida albicans. Exp. Opin. Pharmacother..

[187] Nijveldt R.J., van Nood E., van Hoorn D.E.C., Boelens P.G., van Norren K., van Leeuwen P.A.M. (2001). Flavonoids: a review of probable mechanisms of action and potential applications. Am. J. Clin. Nutr..

[189] Oh B., Figtree G., Costa D., Eade T., Hruby G., Lim S., Elfiky A., Martine N., Rosenthal D., Clarke S., Back M. (2016). Oxidative stress in prostate cancer patients: a systematic review of case control studies. Prostate Int..

[190] Ohsaki A., Takashima J., Chiba N., Kawamura M. (1999). Microanalysis of a selective potent anti-Helicobacter pylori compound in a Brazilian medicinal plant, Myroxylon peruiferum and the activity of analogues. Bioorg. Med. Chem. Lett..

[191] Olaleye S.B., Farombi E.O. (2006). Attenuation of indomethacin‐and HCl/ethanol‐induced oxidative gastric mucosa damage in rats by kolaviron, a natural biflavonoid of Garcinia kola seed. Phytother. Res. Int. J. Dev. Pharmacol. Toxicol. Eval. Nat. Prod. Deriv..

[192] Oliveira M.Rd, Nabavi S.F., Daglia M., Rastrelli L., Nabavi S.M. (2016). Epigallocatechin gallate andmitochondria—A story of life and death. Pharmacol. Res..

[193] Oliveira V.M., Carraro E., Auler M.E., Khalil N.M. (2016). Quercetin and rutin as potential agents antifungalagainst Cryptococcus spp. Braz. J. Biol.

[194] Omosa L.K., Midiwo J.O., Mbaveng A.T., Tankeo S.B., Seukep J.A., Voukeng I.K., Omolle R.A. (2016). Antibacterial activities and structure–activity relationships of a panel of 48 compounds from Kenyan plants against multidrug resistant phenotypes. Springer Plus.

[195] Omosa L.K., Midiwo J.O., Mbaveng A.T., Tankeo S.B., Seukep J.A., Voukeng I.K., Omolle R.A. (2016). Antibacterial activities and structure–activity relationships of a panel of 48 compounds from Kenyan plants against multidrug resistant phenotypes. Springer Plus.

[196] Orallo F., Álvarez E., Basaran H., Lugnier C. (2004). Comparative study of the vasorelaxant activity, superoxide-scavenging ability and cyclic nucleotide phosphodiesterase-inhibitory effects of hesperetin and hesperidin. Naunyn-Schmiedeberg’s Arch. Pharmacol..

[197] Orhan D.D., Özçelik B., Özgen S., Ergun F. (2010). Antibacterial, antifungal, and antiviral activities of some flavonoids. Microbiol. Res..

[198] Pan Y., K L., Li Y.C. (2007). icariin from Epimedium brevicornum attenuates chronic mild stress-induced behavioral and neuroendocrinological alterations in male Wistar rats. Pharmacol. Biochem. Behav..

[199] Park J.Y., Yuk H.J., Ryu H.W., Lim S.H., Kim K.S., Park K.H., Ryu Y.B., Lee W.S. (2017). Evaluation of polyphenols from Broussonetia papyrifera as coronavirus protease inhibitors. J. Enzyme Inhib. Med. Chem..

[200] Park M.H., Ju J.W., Kim M., Han J.S. (2016). The protective effect of daidzein on high glucose-induced oxidative stress in human umbilical vein endothelial cells. Zeitschrift Naturforschung C.

[201] Pasetto S., Pardi V., Murata R.M. (2014). Anti-HIV-1 activity of flavonoid myricetin on HIV-1 infection in a dual-chamber in vitro model. PLoS One.

[202] Patel K.R., Brown V.A., Jones D.J., Britton R.G., Hemingway D., Miller A.S. (2010). Clinical pharmacology of resveratrol and its metabolites in colorectal cancer patients. Cancer Res..

[203] Paulke A., N M., Schubert-Zsilavecz M. (2008). St. John's wort flflavonoids and their metabolites show antidepressant activity and accumulate in brain after multiple oral doses. Pharmazie.

[204] Paulo L., Oleastro M., Gallardo E., Queiroz J.A., Domingues F. (2011). Anti-Helicobacter pylori and urease inhibitory activities of resveratrol and red wine. Food Res. Int..

[205] Peralta M.A., Ortega M.G., Cabrera J.L., Paraje M.G. (2018). The antioxidant activity of a prenyl flavonoid alters its antifungal toxicity on Candida albicans biofilms. Food Chem. Toxicol..

[206] Philips B.J., Coyle C.H., Morrisroe S.N., Chancellor M.B., Yoshimura N. (2009). Induction of apoptosis in human bladder cancer cells by green tea catechins. Biomed. Res..

[207] Pietta P.G. (2000). Flavonoids as antioxidants. J. Nat. Prod..

[208] Plundrich N.J., Bansode R.R., Foegeding E.A., Williams L.L., Lila M.A. (2017). Protein-bound Vaccinium fruit polyphenols decrease IgE binding to peanut allergens and RBL-2H3 mast cell degranulation in vitro. Food Funct..

[209] Prior R.L., Cao G. (2000). Antioxidant phytochemicals in fruits and vegetables: diet and health implications. Hortic. Sci..

[210] Procházková D., Boušová I., Wilhelmová N. (2011). Antioxidant and prooxidant properties of flavonoids. Fitoterapia.

[211] Qamar M.T., Alqahtani S.M., Alamri M.A., Chen L.L. (2020). Structural basis of SARS-CoV-2 3CLpro and anti-COVID-19 drug discovery from medicinal plants. J. Pharm. Anal..

[212] Qamar M.T., Alqahtani S.M., Alamri M.A., Chen L.L. (2020). Structural basis of SARS-CoV-2 3CLpro and anti-COVID-19 drug discovery from medicinal plants. J. Pharm. Anal..

[213] Qian S., Fan W., Qian P., Zhang D., Wei Y., Chen H., Li X. (2015). Apigenin restricts FMDV infection and inhibitsviral IRES driven translational activity. Viruses.

[214] Qiao Y., Cao J., Xie L., Shi X. (2009). Cell growth inhibition and gene expression regulation by (-)-epigallocatechin-3-gallate in human cervical cancer cells. Arch. Pharm. Res..

[215] Quílez A., Berenguer B., Gilardoni G., Souccar C., De Mendonça S., Oliveira L.F.S., Martín-Calero M.J., Vidari G. (2010). Anti-secretory, anti-inflammatory and anti-Helicobacter pylori activities of several fractions isolated from Piper carpunya Ruiz & Pav. J. Ethnopharmacol..

[217] Rahardiyan D. (2019). Antibacterial potential of catechin of tea (Camellia sinensis) and its applications. Food Res..

[218] Rajadurai M., Prince P.S.M. (2007). Preventive effect of naringin on isoproterenol-induced cardiotoxicity in Wistar rats: an in vivo and in vitro study. Toxicology.

[220] Ramadan M.A., Safwat N.A. (2009). Antihelicobacter activity of a flavonoid compound isolated from Desmostachya bipinnata. Aust. J. Bas. Appl. Sci..

[221] Ravishankar D., Salamah M., Akimbaev A., Williams H.F., Albadawi D.A., Vaiyapuri R., Greco F., Osborn H.M., Vaiyapuri S. (2018). Impact of specific functional groups in flavonoids on the modulation of platelet activation. Sci. Rep..

[222] Ravishankar D., Salamah M., Attina A., Pothi R., Vallance T.M., Javed M., Williams H.F., Alzahrani E.M., Kabova E., Vaiyapuri R., Shankland K. (2017). Ruthenium-conjugated chrysin analogues modulate platelet activity, thrombus formation and haemostasis with enhanced efficacy. Sci. Rep..

[223] Reis M.P.C., Carvalho C.R.C., Andrade F.A., Fernandes O.D.F.L., Arruda W., Silva M.R.R. (2016). Fisetin as a promising antifungal agent against Cryptocococcus neoformans species complex. J. Appl. Microbiol..

[226] Ruggiero P., Rossi G., Tombola F., Pancotto L., Lauretti L., Del Giudice G., Zoratti M. (2007). Red wine and green tea reduce H pylori-or VacA-induced gastritis in a mouse model. World J. Gastroenterol..

[227] Russo M., Moccia S., Spagnuolo C., Tedesco I., Russo G.L. (2020). Roles of flavonoids against coronavirus infection. Chem.-biol Interact..

[228] Ryu S., Lim W., Bazer F.W., Song G. (2017). Chrysin induces death of prostate cancer cells by inducing ROS and ER stress. J. Cell Physiol..

[229] Saito H., Tamura M., Imai K., Ishigami T., Ochiai K. (2013). Catechin inhibits Candida albicans dimorphism bydisrupting Cek1 phosphorylation and cAMP synthesis. Microb. Pathogen..

[230] Salam M.A., Ibrahim B.M., El-Batran S.E., El-Gengaihi S.E., Abou Baker D.H. (2016). Study of the possible antihypertensive and hypolipidemic effects of an herbal mixture on l-name-induced hypertensive rats. Asian J. Pharm. Clin. Res..

[231] Salas M.P., Céliz G., Geronazzo H., Daz M., Resnik S.L. (2011). Antifungal activity of natural and enzymatically-modified flavonoids isolated from citrus species. Food Chem..

[234] San Tang K. (2020). The potential role of nanoyttria in alleviating oxidative stress biomarkers: implications for Alzheimer’s disease therapy. Life Sci..

[235] Saponara S., Testai L., Iozzi D., Martinotti E., Martelli A., Chericoni S., Sgaragli G., Fusi F., Calderone V. (2006). (+/−)‐Naringenin as large conductance Ca2+‐activated K+ (BKCa) channel opener in vascular smooth muscle cells. Br. J. Pharmacol..

[236] Satoh H., Nishida S. (2014). Cardio-electopharmacology and vasodilating mechanisms of quercetin. Med. Chem..

[237] Schwarz S., Wang K., Yu W., Sun B., Schwarz W. (2011). Emodin inhibits current through SARS-associated coronavirus 3a protein. Antivir. Res..

[238] Seleem D., Benso B., Noguti J., Pardi V., Murata R.M. (2016). In vitro and in vivo Antifungal Activity ofLichochalcone-A against Candida albicans Biofilms. PLoS One.

[239] Semwal D.K., Semwal R.B., Combrinck S., Viljoen A. (2016). Myricetin: a dietary molecule with diverse biological activities. Nutrients.

[240] Seo H.S., Jo J.K., Ku J.M., Choi H.S., Choi Y.K. (2015). Induction of caspase dependent extrinsic apoptosis through inhibition of signal transducer and activator of transcription 3 (STAT3) signaling in HER2-overexpressing BT-474 breast cancer cells. Biosci. Rep..

[241] Serpa R., Franca E.J.G., Furlaneto-Maia L., Andrade C.G.T.J., Diniz A., Furlaneto M.C. (2012). In vitro antifungalactivity of the flavonoid baicalein against Candida species. J. Med. Microbiol..

[242] Shakya T., Stogios P.J., Waglechner N., Evdokimova E., Ejim L., Blanchard J.E., McArthur A.G., Savchenko A., Wright G.D. (2011). A small molecule discrimination map of the antibiotic resistance kinome. Chem. Biol..

[243] Shibata C., Ohno M., Otsuka M. (2014). The flavonoid apigenin inhibits hepatitis C virus replication by decreasing mature microRNA122 levels. Virology.

[244] Singh A., Demont A., Actis-Goretta L., Holvoet S., Lévêques A., Lepage M., Nutten S., Mercenier A. (2014). Identification of epicatechin as one of the key bioactive constituents of polyphenol-enriched extracts that demonstrate an anti-allergic effect in a murine model of food allergy. Br. J. Nutr..

[245] Sloley B.D.U., L J., Morley P., Durkin J., Shan J.J., Pang P.K.T., Coutts R.T. (2000). Identification of kaempferol as a monoamine oxidase inhibitor and potential neuroprotectant in extracts of Ginkgo biloba leaves. J. Pharm. Pharmacol..

[246] Song X., Zhou B., Zhang P., Lei D., Wang Y., Yao G., Hayashi T., Xia M., Tashiro S.I., Onodera S., Ikejima T. (2016). Protective effect of silibinin on learning and memory impairment in LPS-Treated Rats via ROS–BDNF–TrkB Pathway. Neurochem. Res..

[247] Souza L.C., d G.M., Goes A.T. (2013). Evidence for the involvement of the serotonergic 5- HT (1A) receptors in the antidepressant-like effect caused by hesperidin in mice. Prog. Neuro-Psychopharmacol. Biol. Psychiatry.

[248] Sugasawa N., Katagi A., Kurobe H., Nakayama T., Nishio C., Takumi H., Higashiguchi F., Aihara K.I., Shimabukuro M., Sata M., Kitagawa T. (2019). Inhibition of atherosclerotic plaque development by oral administration of α-glucosyl hesperidin and water-dispersible hesperetin in apolipoprotein E knockout mice. J. Am. Coll. Nutr..

[249] Sumbul S., Ahmad M.A., Mohd A., Mohd A. (2011). Role of phenolic compounds in peptic ulcer: an overview. J. Pharmacy Bioallied Sci..

[250] Sun L., Liao K., Wang D. (2015). E_ects of magnolol and honokiol on adhesion, yeast-hyphal transition, andformation of biofilm by Candida albicans. PLoS One.

[251] Takabayashi F., Harada N., Yamada M., Murohisa B., Oguni I. (2004). Inhibitory effect of green tea catechins in combination with sucralfate on Helicobacter pylori infection in Mongolian gerbils. J. Gastroenterol..

[252] Takumi H., Nakamura H., Simizu T., Harada R., Kometani T., Nadamoto T., Mukai R., Murota K., Kawai Y., Terao J. (2012). Bioavailability of orally administered water-dispersible hesperetin and its effect on peripheral vasodilatation in human subjects: implication of endothelial functions of plasma conjugated metabolites. Food Funct..

[253] Teissedre P.L., Frankel E.N., Waterhouse A.L., Peleg H., German J. Bruce (1996). Inhibition of in vitro human LDL oxidation by phenolic antioxidants from grapes and wines. J. Sci. Food Agric..

[254] Thorn C.F., Oshiro C., Marsh S., Hernandez-Boussard T., McLeod H., Klein T.E. (2011). Doxorubicin pathways: pharmacodynamics and adverse effects. Pharmacogenet. Genom..

[257] Tsou C.H., Lee H.T., Hung W.S., Wang C.C., Shu C.C., Suen M.C., De Guzman M. (2016). Synthesis and properties of antibacterial polyurethane with novel Bis (3-pyridinemethanol) silver chain extender. Polymer.

[258] Tutunchi H., Naeini F., Ostadrahimi A., Hosseinzadeh‐Attar M.J. (2020). Naringenin, a flavanone with antiviral and anti‐inflammatory effects: A promising treatment strategy against COVID‐19. Phytother. Res..

[259] Ustün O., Ozçelik B., Akyön Y., Abbasoglu U., Yesilada E. (2006). Flavonoids with anti-Helicobacter pylori activity from Cistus laurifolius leaves. J. Ethnopharmacol..

[260] Vaiyapuri S., Roweth H., Ali M.S., Unsworth A.J., Stainer A.R., Flora G.D. (2015). Pharmacological actions of nobiletin in the modulation of platelet function. Br. J. Pharmacol..

[262] van Acker S.A., Tromp M.N., Haenen G.R., van der Vijgh W.J., Bast A. (1995). Flavonoids as scavengers of nitric oxide radical. Biochem. Biophys. Res. Commun..

[263] Varoni E.M., Faro A.F.L., Sharifi-Rad J., Iriti M. (2016). Anticancer molecular mechanisms ofresveratrol. Front. Nutr..

[264] Vijayakumar B.G., Ramesh D., Joji A., Kannan T. (2020). In silico pharmacokinetic and molecular docking studies of natural flavonoids and synthetic indole chalcones against essential proteins of SARS-CoV-2. Eur. J. Pharmacol..

[265] Vijayakumar B.G., Ramesh D., Joji A., Kannan T. (2020). In silico pharmacokinetic and molecular docking studies of natural flavonoids and synthetic indole chalcones against essential proteins of SARS-CoV-2. Eur. J. Pharmacol..

[266] Villeneuve L., Girard H., Fortier L.C., Gagné J.F., Guillemette C. (2003). Novel functional polymorphisms in the UGT1A7 and UGT1A9 glucuronidating enzymes in Caucasian and African-American subjects and their impact on the metabolism of 7-ethyl-10-hydroxycamptothecin and flavopiridol anticancer drugs. J. Pharmacol. Exp. Ther..

[267] Kim W.Y., Son M., Ko J.I., Cho H., Yoo M., Kim W.B., Song I.S., Kim C.Y. (1999). Arch. Pharm. Res..

[268] Wallace T.C., Slavin M., Frankenfeld C.L. (2016). Systematic review of anthocyanins and markers of cardiovascular disease. Forum Nutr..

[270] Wang D., Sun Q., Wu J., Wang W., Yao G., Li T., Li X., Li L., Zhang Y., Cui W. (2017). A new PrenylatedFlavonoid induces G0/G1 arrest and apoptosis through p38/JNK MAPK pathways in Human HepatocellularCarcinoma cells. Sci. Rep..

[271] Wang H.H., Chung J.G. (1997). Emodin-induced inhibition of growth and DNA damage in the Helicobacter pylori. Curr. Microbiol..

[272] Wang Q., Xie M. (2010). Antibacterial activity and mechanism of luteolin on Staphylococcus aureus. Wei sheng wu xue bao= Acta Microbiol. Sin..

[275] Wasowski C., Marder M. (2012). Flavonoids as GABAA receptor ligands: the whole story?. J. Exp. Pharmacol..

[279] Wittschier N., Faller G., Hensel A. (2009). Aqueous extracts and polysaccharides from liquorice roots (Glycyrrhiza glabra L.) inhibit adhesion of Helicobacter pylori to human gastric mucosa. J. Ethnopharmacol..

[280] Wood P.J. (1986). Oat β-glucan: structure, location and properties. Oats: Chem. Technol..

[281] Wu P.H., Shen Y.C., Wang Y.H., Chi C.W., Yen J.C. (2006). Baicalein attenuates methamphetamine-induced loss of dopamine transporter in mouse striatum. Toxicology.

[282] Wu Y., Wang Y., Nabi X. (2019). Protective effect of Ziziphora clinopodioides flavonoids against H2O2-induced oxidative stress in HUVEC cells. Biomed. Pharmacother..

[283] Xian Y., Zhang J., Bian Z., Zhou H., Zhang Z., Lin Z., Xu H. (2020). Bioactive natural compounds against human coronaviruses: a review and perspective. Acta Pharm. Sin. B.

[284] Xie Y., Yang W., Chen X., Xiao J. (2014). Inhibition of flavonoids on acetylcholine esterase: binding and structure–activity relationship. Food Funct..

[285] Xie Y., Yang W., Tang F., Chen X., Ren L. (2015). Antibacterial activities of flavonoids: Structure‐activity relationship and mechanism. Curr. Med. Chem..

[288] Yeh S.L., Wang W.Y., Huang C.H., Hu M.L. (2005). Pro-oxidative effect of β-carotene and the interaction with flavonoids on UVA-induced DNA strand breaks in mouse fibroblast C3H10T1/2 cells. J. Nutr. Biochem..

[289] Yeşilada E., Gürbüz I., Shibata H. (1999). Screening of Turkish anti-ulcerogenic folk remedies for anti-Helicobacter pylori activity. J. Ethnopharmacol..

[290] Yi L.T., L C.F., Zhan X., Cui C.C., Xiao F., Zhou L.P., Xie Y., Prog Neuropsychopharmacol Biol (2010). Involvement of monoaminergic system in the antidepressant-like effffect of the flflavonoid naringenin in mice. Prog. NeuroPsychopharmacol. Biol. Psychiatry.

[291] Yi L.T., X H., Feng J. (2011). Involvement of monoaminergic systems in the antidepressant-like effect of nobiletin. Physiol. Behav..

[292] Yi L.T.1, L J., Li Y.C., Pan Y., Xu Q., Kong L.D. (2008). Antidepressant-like behavioural and neurochemical effects of the citrus-associated chemical apigenin. Life Sci..

[293] Yigit D., Yigit N., Mavi A. (2009). Antioxidant and antimicrobial activities of bitter and sweet apricot(Prunusarmeniaca L.) kernels. Braz. J. Med. Biol. Res..

[294] Zaidi S.F.H., Yamada K., Kadowaki M., Usmanghani K., Sugiyama T. (2009). Bactericidal activity of medicinal plants, employed for the treatment of gastrointestinal ailments, against Helicobacter pylori. J. Ethnopharmacol..

[295] Zakaryan H., Arabyan E., Oo A., Zandi K. (2017). Flavonoids: promising natural compounds against viral infections. Arch. Virol..

[296] Zhang J., Wu Y., Zhao X. (2014). Chemopreventive effect of flavonoids from Ougan (Citrus reticulata cv. Suavissima) fruit against cancer cell proliferation and migration. J. Funct. Foods.

[297] Zhang X., Huang H., Zhao X. (2015). Effects of flflavonoids-rich Chinese bayberry (Myrica rubra Sieb. et Zucc.) pulp extracts on glucose consumption in human HepG2 cells. J. Funct. Foods.

[298] Zhang F., Wang Y.Y., Liu H., Lu Y.F., Wu Q., Liu J., Shi J.S. (2012). Resveratrol produces neurotrophic effects on cultured dopaminergic neurons through prompting astroglial BDNF and GDNF release. Evid.-based Complement. Altern. Med..

[299] Zhang Q.J., Z Y., Yang M. (2001). The effect of flavonoids on central nervous system. Zhongguo Zhongyao Zazhi.

[300] Zhang Y., Liao P., Li W., Hu D., Chen L., Guan S. (2017). Baicalin attenuates cardiac dysfunction and myocardial remodeling in a chronic pressure-overload mice model. Cell. Physiol. Biochem..

[301] Zhao Y., Dang M., Zhang W., Lei Y., Ramesh T., Veeraraghavan V.P., Hou X. (2020). Neuroprotective effects of Syringic acid against aluminium chloride induced oxidative stress mediated neuroinflammation in rat model of Alzheimer’s disease. J. Funct. Foods.

[302] Zheng M.1, L C., Pan F., Shi D., Zhang Y. (2012). Antidepressant-like effffect of hyperoside isolated from Apocynum venetum leaves: possible cellular mechanisms. Phytomedicine.

[303] Zhu J.T., Choi R.C., Chu G.K., Cheung A.W., Gao Q.T., Li J., Jiang Z.Y., Dong T.T., Tsim K.W. (2007). Flavonoids possess neuroprotective effffects on cultured pheochromocytoma PC12 cells: a comparison of difffferent flflavonoids in activating estrogenic effect and in preventing β-amyloid-induced cell death. J. Agric. Food Chem..

[304] Zhu J.T., Choi R.C., Chu G.K., Cheung A.W., Gao Q.T., Li J., Jiang Z.Y., Dong T.T., Tsim K.W. (2007). Flavonoids possess neuroprotective effects on cultured pheochromocytoma PC12 cells: a comparison of different flavonoids in activating estrogenic effect and in preventing β-amyloid-induced cell death. J. Agric. Food Chem..

[305] Zuzarte M., Vale-Silva L., Gonçalves M.J., Cavaleiro C., Vaz S., Canhoto J., Pinto E., Salgueiro L. (2011). Antifungal activity of phenolic-rich Lavandulamultifida L. essential oil. Eur. J. Clin. Microbiol. Infect. Dis..

